# Stable microbial community promotes aerobic methanotrophy in a large river-reservoir system

**DOI:** 10.1128/msystems.00530-25

**Published:** 2025-06-25

**Authors:** Qiong Tang, Zhe Li, Lunhui Lu, Yan Xiao, Xinghua Wu, Dianchang Wang

**Affiliations:** 1 Chongqing Jiaotong University 47912, Chongqing, China; 2 CAS Key Lab of Reservoir Environment, Chongqing Institute of Green and Intelligent Technology, Chinese Academy of Sciences 376348, Chongqing, China; 3 College of Resources and Environment, Chongqing School, University of Chinese Academy of Sciences, Chongqing, China; 4 National Engineering Research Center of Eco-Environment in the Yangtze River, China Three Gorges Corporation 127240, Beijing, China; University of East Anglia, Norwich, United Kingdom

**Keywords:** CH_4_, methane-derived carbon, hydrological gradient, methane-oxidizing bacteria, community stability

## Abstract

Aerobic methanotrophy, mainly carried out by methane-oxidizing bacteria (MOB) in freshwater systems, plays a crucial role in reducing methane (CH_4_) emissions and serves as an additional carbon source (methane-derived carbon) that supports the development of microbial food loops. However, how hydrological gradients and trophic states jointly regulate methane-derived carbon dynamics and microbial community stability in river-reservoir systems, and the interplay between these two aspects, has not been adequately explored. Here, we analyze bacterial and microeukaryotic organisms using 16S rRNA and 18S rRNA gene sequencing, respectively, in a river-reservoir complex in the upper Yangtze River basin. Our results show that species diversity and interaction strength with MOB are key to maintaining community stability in the riverine zone. In addition, the more pronounced niche differentiation also contributes to community stability in the lacustrine zone, which in turn facilitates the function of aerobic methanotrophy. In the riverine and lacustrine zones, the keystone species shift from the Alphaproteobacteria class (Alpha-MOB) to the Gammaproteobacteria class (Gamma-MOB), indicating distinct mechanisms for maintaining community stability in these zones. Moreover, methane-derived carbon in the riverine and lacustrine zones and in the mesotrophic states is crucial for supporting bacterial productivity and is associated with the highest community stability. These insights highlight the complex but important role of aerobic methanotrophy in freshwater systems.

**IMPORTANCE:**

Our study elucidates the ecological underpinnings of aerobic methanotrophy (methane-derived carbon) in freshwater systems, revealing a pivotal role of community stability in modulating aerobic methanotrophy across hydrological gradients. We identify a shift in dominant methane-oxidizing bacteria (MOB) classes from Alpha- to Gammaproteobacteria, highlighting distinct mechanisms for community stability maintenance and methane-derived carbon utilization, crucial for bacterial productivity and ecosystem health. This study enhances our understanding of methane dynamics in freshwater systems, a subject with significant implications for climate change mitigation, and provides valuable insights into the microbial food loop and carbon cycling, aligning with the focus of *mSystems*.

## INTRODUCTION

The pressing issue of climate change and its global ramifications have thrust freshwaters into the spotlight of environmental research. As underscored by Williamson et al. (1), freshwaters serve as sentinels of global warming and play a pivotal role in climate regulation by acting as substantial carbon contributors to the atmosphere. Over the past decades, global freshwaters have emitted approximately 1.5 Pg C-CO_2_·year^−1^ and 159 Tg CH_4_·year^−1^, as reported by the Intergovernmental Panel on Climate Change Sixth Assessment Report (2). These figures highlight the significant role of freshwaters in global carbon cycling.

Aerobic methanotrophy, performed chiefly by methane-oxidizing bacteria (MOB) in freshwaters, mainly serves a dual purpose: it acts as a “biofilter” or the last “barrier” of CH_4_ from freshwater into the atmosphere (preventing CH_4_ release to the atmosphere) and is an essential part of bacterial productivity in the water column. Both significantly alter the CH_4_ balance between freshwater and the atmosphere (3, 4). MOB is a functional group of bacteria that mainly includes Gammaproteobacteria class (Gamma-MOB, type I), Alphaproteobacteria class (Alpha-MOB, type II), Verrucomicrobia, and NC10 phylum, which utilizes CH_4_ as its only carbon and energy source (5, 6). These heterotrophic bacteria typically account for a minor portion of the microbial community (6, 7), although they can achieve substantial densities within specific oxic/anoxic interfaces (8). However, they appear to have a disproportionately significant impact on shaping the freshwater biogeochemical cycles (9). Crevecoeur et al. (10) reported that the methanotrophic community in the boreal landscape, despite minor regional variations, was strongly influenced by the hydrologic continuum, reflecting a distinct niche for type I and type II MOB. Some researchers showed that Gamma-MOB is usually dominant in temperate lakes, such as *Methylobacter* and *Methylosoma* (7, 11). Although recent advances increase our understanding of the biogeographical distributions of MOB, the functioning of aerobic methanotrophy in freshwaters could still be challenging to predict due to the eco-physiological response of MOB under changing environmental conditions, shaping the non-linearity of biodiversity-ecosystem functioning relationships (12). For instance, Thottathil et al. (13) reported that sustained high oxygen in the water column may inhibit MOB activity. Although MOB diversity does not directly correlate with aerobic methanotrophy (particularly in dormant states), their persistent presence nourishes the microbial food loop through biomass provision and maintains functional resilience via a metabolically versatile seed bank that buffers against environmental fluctuations (14). Therefore, the causal effects between MOB diversity and the functioning of aerobic methanotrophy and their driving factors are not well comprehended.

Concurrently, the utilization of CH_4_ by MOB created methane-derived carbon, which flows into the microbial food loop and acts as a crucial carbon source in freshwaters (15, 16). For instance, methane-derived carbon can be utilized directly or indirectly by zooplankton (17, 18). This fuels primary and secondary productivity and supports the development and succession of microbial food loops with “MOB-zooplankton or benthonic,” profoundly affecting the structure and function of aquatic systems (16, 19). Additionally, it was reported to contribute more to some invertebrates during algal blooms, becoming an alternative source of photosynthesis-derived organic carbon (16). In some stratified lakes, methane-derived carbon can sustain up to 80% of overall bacterial productivity (15, 19, 20). Thus, methane-derived carbon and its share are believed to be important indicators of the contribution of aerobic methanotrophy to microbial food loops. The fate and abundance of methane-derived carbon in the water column are related to the community structure of MOB. Schilder et al. (16) reported that daphnia supports their growth through the assimilation of Gamma-MOB. Most existing studies highlighted the importance of methane-derived carbon as an indispensable carbon source from the organism’s perspective that benefits from it (15, 16, 21). However, the question of how the ecology of MOB in the microbial community, through the provision of methane-derived carbon, structured the microbial food loop in the freshwater system is still not well addressed.

Community stability has emerged recently as a critical framework for understanding the microbiology dynamics in freshwater (22, 23). Community stability encapsulates the ability of biological communities to withstand and recover from external disturbances (24). Influenced by environmental factors like temperature, pH, and nutrient availability, microbial interactions such as predation, competition, and symbiosis can modulate community stability (25, 26). Yuan et al. (26) revealed that experimental warming significantly enhances the complexity of soil microbial ecological networks, positively correlated with different community stability indices, supporting the ecological principle that complexity begets stability. Microbial communities that exhibit greater stability seemed more likely to enhance ecosystem functions, such as carbon degradation (26). Li et al. (27) showed that night lighting disrupts soil bacteria, making the network unstable and changing the denitrification potential. The implications of these discoveries are profound, as they imply that stable microbial communities are prerequisites for supporting their specific ecosystem functions and microbial interactions. Crucially, this stability directly governs aerobic methanotrophy through these interdependent mechanisms. For example, stable communities maintain diverse MOB assemblages (e.g., Gamma-MOB dominate in hypoxic zones, Alpha-MOB in oxic niches), ensuring that aerobic methanotrophy persists even if taxa decline (28). This redundancy prevents functional collapse during perturbations. Moreover, community stability may modulate the interaction relationship between MOB and heterotrophs through cross-feeding of organic carbon or other nutrients (29). Additionally, community stability supports aerobic methanotrophy by maintaining the activity of bacteria, regulating interspecific interactions, and buffering environmental stresses; conversely, efficient aerobic methanotrophy enhances community stability by regulating the microenvironment and resource allocation. This bi-directional relationship is an important mechanism for the regulation of ecosystem functioning and is particularly important for the prediction and management of CH_4_ cycling in the context of global change. However, whether community stability under hydrological and trophic gradients can promote the functioning of aerobic methanotrophy remains unclear.

Dam construction and reservoir creation have transformed rivers from their natural, free-flowing state into regulated water bodies for various purposes (30). These newly created habitats, formed by the hydrological gradients, have been recognized for significantly impacting aquatic productivity and the composition and function of microbial communities. This, in turn, contributes to the cycling of biogenic elements in river-reservoir systems (31, 32). Analyzing river-reservoir systems through the hydrological or trophic gradient provides a framework for understanding aerobic methanotrophy and its role in community stability. The scale and heterogeneity of river-reservoir systems pose challenges for comprehensive sampling and data interpretation, leading to an underestimation of the spatiotemporal variability for MOB. Therefore, investigating the influence of MOB on community stability presents a substantial research interest.

Here, the river-reservoir complex in the upper Yangtze River basin serves as a typical example. Despite growing recognition of microbial community stability as a regulator of ecosystem functions, critical gaps persist in understanding its role in methane oxidation dynamics, especially along the hydrological gradient and trophic gradient. Moreover, the perspective of characterizing aerobic methanotrophy in terms of methane-derived carbon is rarely reported. Therefore, we assume that species with highly specialized metabolic capacities, such as MOB, may substantially affect community stability through interaction with zooplankton, thereby contributing to the production of methane-derived carbon and species diversity. Specifically, we hypothesize that (i) MOB diversity and interaction strength will positively contribute to community stability and (ii) community stability may also promote the production of methane-derived carbon, thereby supporting MOB diversity through positive feedback. This study endeavors to advance our understanding of community stability and its contribution to carbon cycling in river-reservoir systems, potentially informing strategies for managing CH_4_ emissions and preserving aquatic biodiversity.

## MATERIALS AND METHODS

### Experimental design

**Fig 1:**
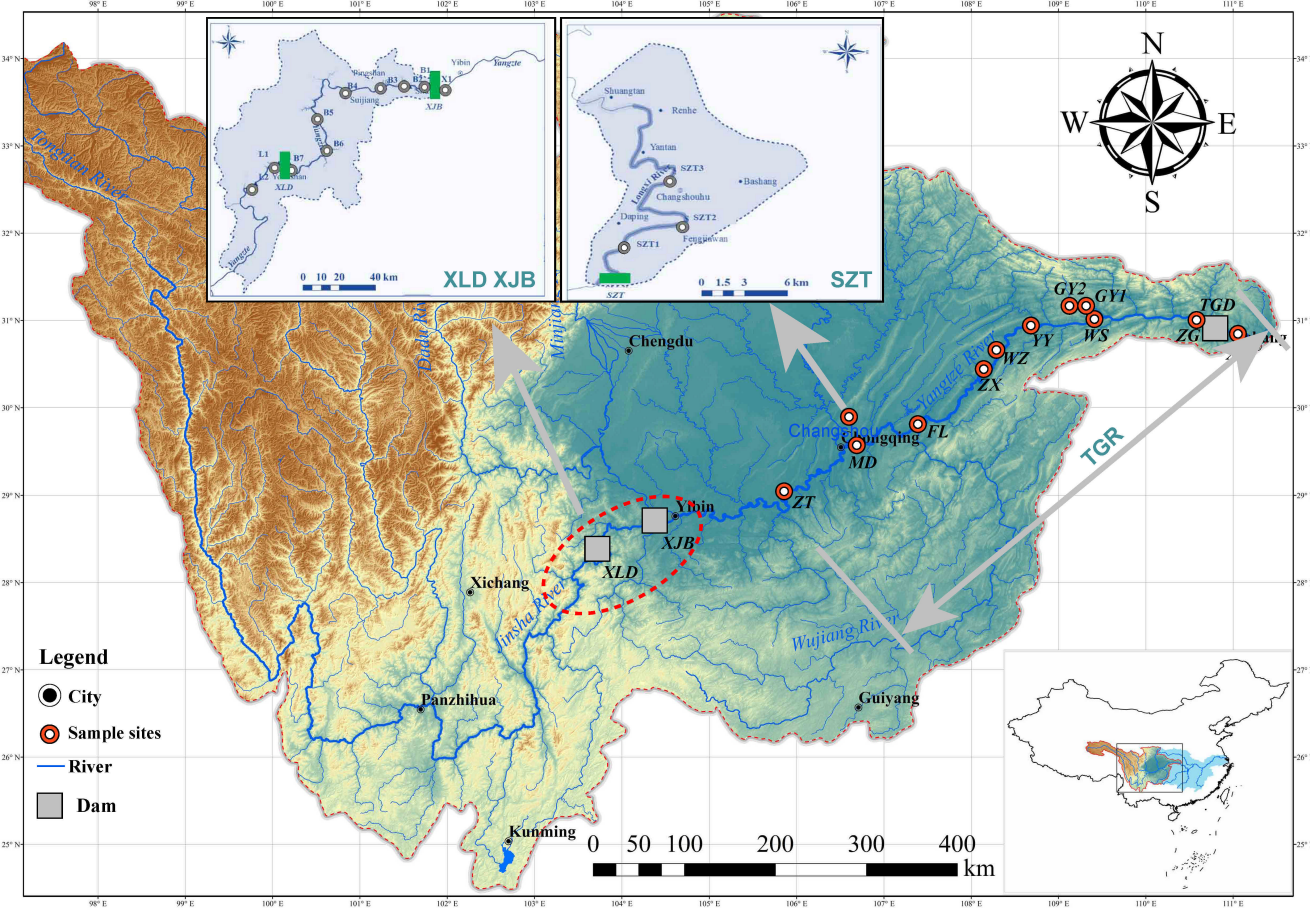
A map depicting the sampling sites located in the upper Yangtze River (adapted from reference 33). The area selected for the TGR is from Zhutuo (ZT) to Sandouping (SDP). Xiluodu (XLD), Xiangjiaba (XJB), and Shizitan (SZT) reservoir catchments associated with sampling sites are amplified on the map.

In the study, we chose four large reservoirs in the upper Yangtze River basin, as depicted in Fig. 1, namely, Xiluodu Reservoir (XLD; 27°12′54.9″–28°15′22.30″N, 102°54′15.94″–103°39′11.19″E), Xiangjiaba Reservoir (XJB; 28°15′17.52″–28°38′29.82″N, 103°39′10.18″–104°23′41.48″E), Three Gorges Reservoir (TGR; 28°46′15.71″–30°49′23.03″N, 104°38′5.62″–111°0′11.82″E), and Shizitan Reservoir (SZT; 29°54′21.98″N, 107°14′53.79″E). XLD, XJB, and TGR are large cascading river-valley reservoirs on the main course of the Yangtze River. The XLD and XJB were formed in 2012 and 2013, respectively. The initial impoundment of the TGR was in 2003. In 2010, the TGR first reached its normal operational water level, i.e., 175 m above the sea. SZT was an “old” dendritic reservoir formed in 1955. The parameters of the four selected reservoirs are shown in Table S1.

Twenty-four sampling sites in the main channel were established in these four reservoirs: two in XLD, eight in XJB, eleven in TGR, and three in SZT (Table S1; Fig. 1). All sampling sites were conducted in May, July, and November 2019, and we combined these samples to expand the sample size. Three replicates were set for each sample. The vertical profile in the pre-dam area is shown in Fig. S1. Approximately 10 L of water samples were collected in different vertical layers at each sampling site. In all zones, the surface layer, i.e., 0.5 m below the water surface, and the bottom layer, i.e., 1.0 m above the sediment, were sampled. An additional middle layer was sampled in the transitional and lacustrine zone. See “Data compilation and analysis strategy” in the Supplemental material for details.

For bacterial and microeukaryotic eDNA analysis, water samples were immediately filtered through 0.22 µm Millipore cellulose filters (Milford, MA, USA), and the filters were subsequently stored at −86°C for DNA extraction. Also in the field, about 1 L of water was quickly filtered through 1.2 µm Whatman GF/C glass fiber membranes (Whatman, UK), and then the membrane was preserved in the on-board refrigerator at −20°C for chlorophyll *a* (Chl *a*) analysis upon return to the laboratory. The remaining fresh samples (5 L) were preserved in polyethylene containers with 5 mL of 1 mol·L^−1^ HgCl_2_ to inhibit microbial activity and transported to the laboratory for subsequent analysis.

### Environmental parameters

Dissolved oxygen (DO), water temperature (WT), and pH were directly measured on-site using a YSI EXO2 Multiparameter Sonde (Ohio, USA). Chl *a* concentration was determined by extracting samples with Whatman GF/C filters, followed by spectrophotometric analysis after a 36 hour extraction in 90% cool acetone under dark conditions. Different forms of nitrogen (N) and phosphorus (P) were analyzed using a UV spectrophotometer, adhering to standardized methods (34). Both total organic carbon (TOC) and dissolved organic carbon (DOC) were quantified using a Vario TOC analyzer (Elementar, Germany). The concentrations of particulate organic carbon (POC) and particulate organic nitrogen, along with their stable isotopes, were examined using a Thermo Fisher Flash H T Elemental Analyzer for Isotope Ratio MS (Thermo Fisher Scientific, MA, USA). Then, the DOC concentration and *δ*^13^C value of the samples were determined by a total organic carbon analyzer-stable isotope mass spectrometer. The determination of suspended sediment (SPS) involved comparing the weights of the filter membrane before and after drying. The concentration, flux of CH_4_ and CO_2_, and their stable isotopes in the water phase were measured through the headspace method using a G2201-*i* Isotopic Analyzer (Picarro, USA).

### DNA sequencing and bioinformatic analysis

Genomic DNA was extracted in duplicate from the filters using the FastDNA SPIN kit (Mo Bio Laboratories, Inc.). The extracts of duplicate DNA were mixed well for the subsequent PCR amplification. The primers used for the bacterial 16S rRNA gene were 338F and 806R (35). MOB in the subsequent analyses was extracted from the final Amplicon Sequence Variant (ASV) table (10). The 18S rRNA gene of microeukaryotes was amplified using the primers TAReuk454FWD1F and TAReukREV3R (36). Representative sequences for bacteria and microeukaryotes were annotated using the Silva v.138 database (37) and PR2 database (38), respectively. Amplicons were purified with the AxyPrep DNA Gel Extraction Kit (Axygen Biosciences, CA, USA) and sequenced using the Illumina MiSeq PE300 platform (Illumina, San Diego, USA) at the facilities of Shanghai Majorbio Bio-Pharm Technology, Co., Ltd. in Shanghai, China. Although 16S rRNA gene sequencing provides valuable taxonomic insights, its resolution for functional identification (e.g., MOB) remains limited. Therefore, future work will integrate targeted *pmo*A gene or metagenomic analyses to improve the accuracy of MOB classification and validate their ecological interactions.

### Statistics analysis

The alpha diversity, including the richness, Shannon, and Pielou_e (Pielou’s evenness) index, of bacteria and microeukaryotes was analyzed using the vegan package in R (version 4.1.1). Prior to conducting linear regression analyses, we performed Shapiro-Wilk normality tests on all data sets. For variables deviating from normality (*P* < 0.05), Spearman’s rank correlation analysis was subsequently applied to ensure statistical validity. Structural equation modeling (SEM) was employed to dissect the direct and indirect relationships among all variables within the model. The adequacy of the constructed SEMs was determined using Fisher’s *C* values and *P*-values, with models achieving *P*-values >0.05 deemed acceptable. The piecewiseSEM package was utilized for SEM analyses. Community resilience across zones was determined by the similarity measure, calculated as one minus the Bray-Curtis dissimilarity (39). Higher similarity scores, indicating less variability in species composition, suggest greater resilience.

## RESULTS

### Methane-derived carbon supports aquatic productivity

**Fig 2:**
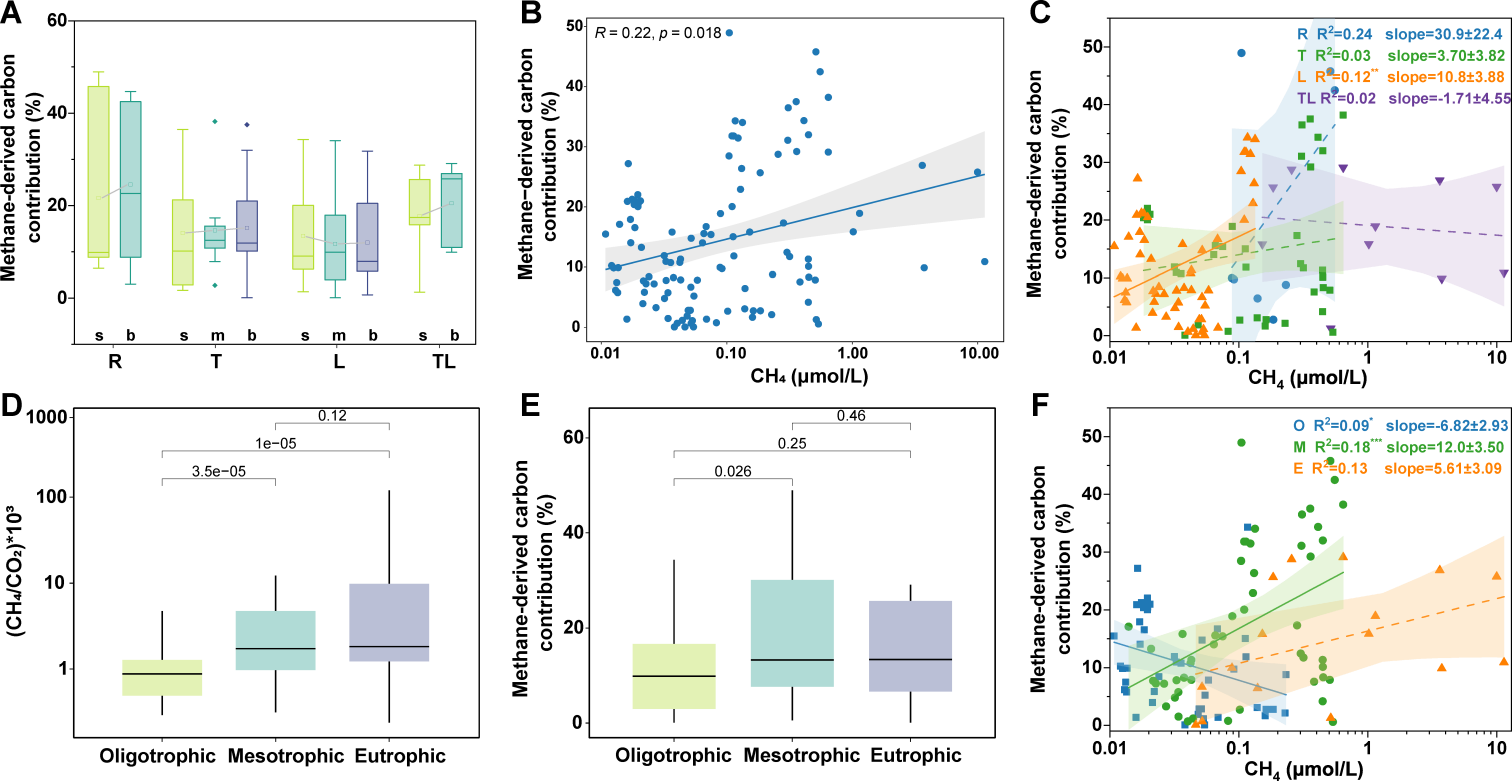
The CH_4_ concentration and contribution of methane-derived carbon variation across the hydrological and trophic gradient. (**A**) Boxplot of the contribution of methane-derived carbon in the surface (s), middle (m), and bottom (b) of the riverine zone (R), transitional zone (T), lacustrine zone (L), and tributary-lacustrine zone (TL). Boxplot hinges indicate the 25th, 50th, and 75th percentiles, whiskers indicate 1.5×  interquartile ranges, and hollow dots denote group means. The sample size is as follows: R_s, 6; R_b, 5; T_s, 24; T_m, 12; T_b, 17; L_s, 27; L_m, 39; L_b, 21; TL_s, 15; TL_b, 15. (**B**) Relationships between CH_4_ concentration and methane-derived carbon. (**C**) Relationships between CH_4_ and methane-derived carbon in the four zones. (**D**) Boxplot of (CH_4_/CO_2_) × 10^3^ in the oligotrophic, mesotrophic, and eutrophic states, respectively. (**E**) Boxplot of methane-derived carbon contribution in the oligotrophic, mesotrophic, and eutrophic states, respectively. In the boxplots of panels **D and E**, hinges indicate the 25th, 50th, and 75th percentiles, and whiskers indicate 1.5×  interquartile ranges. The sample sizes in panels **D and E** are as follows: oligotrophic, 53; mesotrophic, 75; eutrophic, 50. (**F**) The linear relationship between CH_4_ and the contribution of methane-derived carbon in the oligotrophic (O), mesotrophic (M), and eutrophic (**E**) states, respectively. The solid line represented significant relationships (*P* < 0.05), while the dashed line represented non-significant relationships (*P* > 0.05).

Firstly, we found that the contribution of methane-derived carbon to DOC in the river-reservoir system ranges from 0% to 48.9% (Fig. 2A). The vertical profiles across different zones revealed that the contribution of methane-derived carbon slightly elevated with increasing water depth, except in the lacustrine zone (Fig. 2A). In general, the contribution of methane-derived carbon elevated with increasing CH_4_ (Fig. 2B). Specifically, the contribution of methane-derived carbon was positively correlated with CH_4_ concentrations across all zones (excluding TL), with the strongest correlation occurring in the riverine zone (Fig. 2C). In the lacustrine zone, the relationship between CH_4_ concentration and methane-derived carbon contribution remained statistically significant (*P* < 0.05) but exhibited limited explanatory power (*R*^2^ = 0.12), likely reflecting homogenized environmental conditions and low CH_4_ concentration (mean ± SE = 0.05 ± 0.004 µmol/L) (Fig. S1). This suggests that CH_4_ concentration retains a measurable, albeit minor, role in shaping methane-derived carbon assimilation within this zone. It is evident that the higher the concentration of CH_4_, the greater the amount of carbon derived from methane.

Next, we further explored the contribution of methane-derived carbon across the trophic gradient. Initially, we found that the CH_4_/CO_2_ ratio increased with the trophic gradient (Fig. 2D). However, the contribution of methane-derived carbon did not show a consistent increase with the increase of trophic status and showed numerically higher (but statistically non-significant, *P* > 0.05) values under mesotrophic conditions (0.18 ± 0.02) compared to eutrophic states (0.15 ± 0.03) (Fig. 2E). Notably, the contribution of methane-derived carbon significantly increased with an increase in CH_4_ in the mesotrophic states (Fig. 2F).

**Fig 3:**
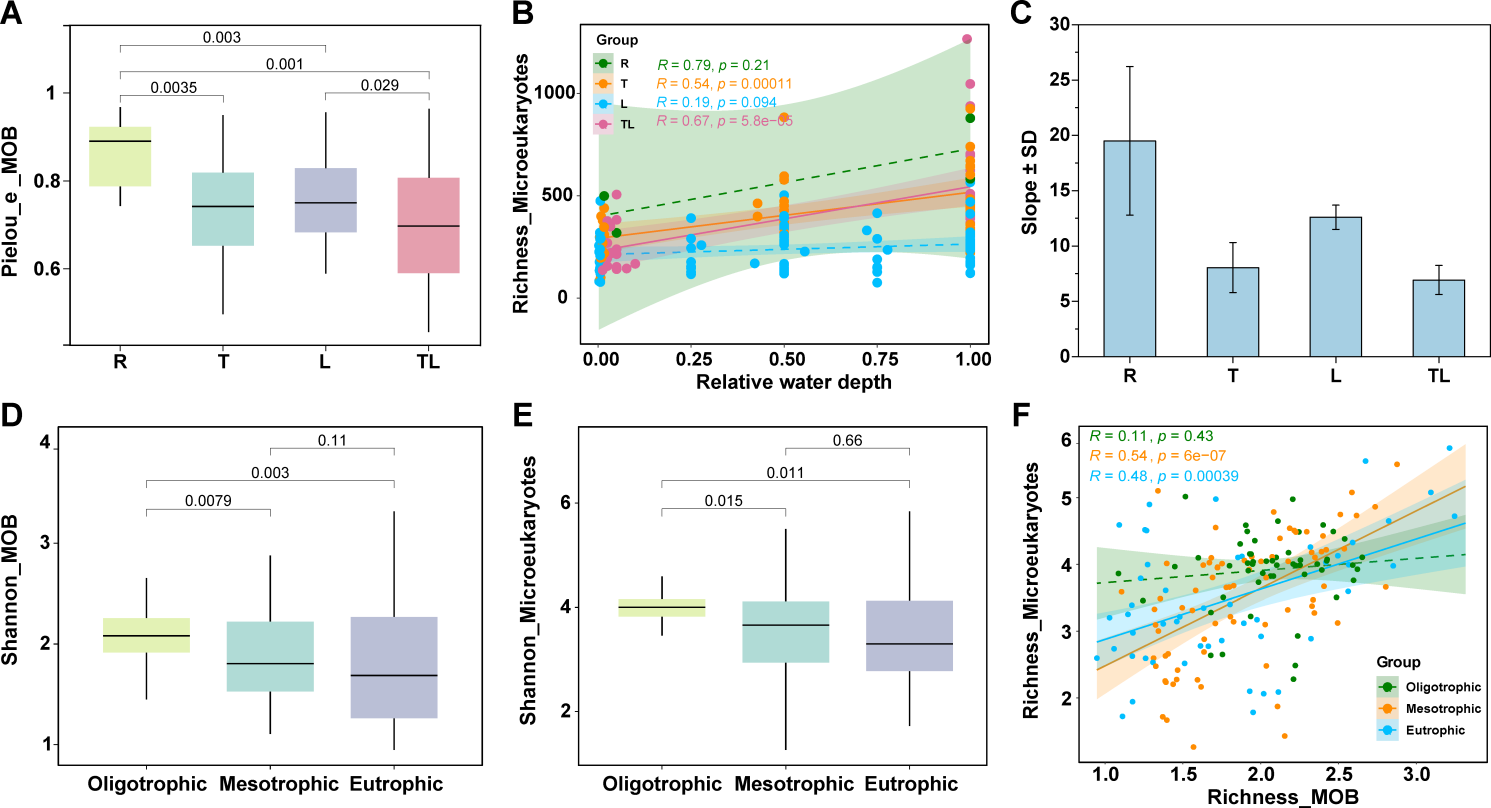
Changes in the α-diversity index for MOB and microeukaryotes both in the hydrological and trophic gradient. (**A**) The Pielou_e (Pielou’s evenness) index of MOB in the riverine zone (R), transitional zone (T), lacustrine zone (L), and tributary-lacustrine zone (TL). (**B**) Trends of richness of microeukaryotes with increasing relative water depth. (**C**) Slope value of richness of MOB correlation analysis with microeukaryotes in the riverine zone (R), transitional zone (T), lacustrine zone (L), and tributary-lacustrine zone (TL). (**D**) Boxplot of Shannon diversity of MOB in the oligotrophic, mesotrophic, and eutrophic states, respectively. (**E**) Boxplot of microeukaryotic community in the oligotrophic, mesotrophic, and eutrophic states, respectively. In the boxplots of panels **A, D, and E**, hinges indicate the 25th, 50th, and 75th percentiles, and whiskers indicate 1.5×  interquartile ranges. (**F**) Correlation relationship between the richness of MOB and microeukaryotic community in the oligotrophic, mesotrophic, and eutrophic states, respectively. The solid line represented significant relationships (*P* < 0.05), while the dashed line represented non-significant relationships (*P* > 0.05).

The Pielou_e (Pielou’s evenness) index of MOB communities exhibited a significant difference along the hydrological gradient (Fig. 3A), with the highest value in the riverine zone (0.84 ± 0.03), significantly exceeding that of the lacustrine zone (0.73 ± 0.01). Transitional (0.70 ± 0.02) and tributary-lacustrine zones (0.67 ± 0.03) showed further reduced index. This indicated that MOB was more evenly distributed in the riverine and lacustrine zones. The richness of microeukaryotes increased with water depth, although not significantly in the riverine zone and lacustrine zone (Fig. 3B). Interestingly, we found that the richness of microeukaryotes increased significantly with MOB regardless of the different hydrological zones (Fig. S2), so we showed their respective slope value separately and found that the slope of MOB vs microeukaryotes was highest in the riverine zone, followed by the lacustrine zone (Fig. 3C). These findings potentially indicate that methane-derived carbon provided by MOB likely constitutes a critical trophic supply for microeukaryotes (referring to zooplankton) communities in both riverine and lacustrine zones. The Shannon diversity of MOB and microeukaryotes did not increase with trophic status, with MOB diversity peaking in the oligotrophic state (2.07 ± 0.05), and significantly decreasing with mesotrophic state (1.89 ± 0.05) vs eutrophic state (1.81 ± 0.09) (Fig. 3D). The diversity distribution patterns of microeukaryotes were similar to those of MOB, with significantly highest values under oligotrophic conditions, while mesotrophic states exhibited slightly higher diversity than eutrophic states numerically, though the difference was not statistically significant (Fig. 3E). In addition, we discovered a significant positive relationship between the richness index of MOB and microeukaryotic community in the mesotrophic sites (Fig. 3F). Consequently, it is apparent that methane-derived carbon by MOB may serve as an important carbon source for zooplankton in the mesotrophic states.

### Biotic interactions of MOB support microbial community stability

**Fig 4:**
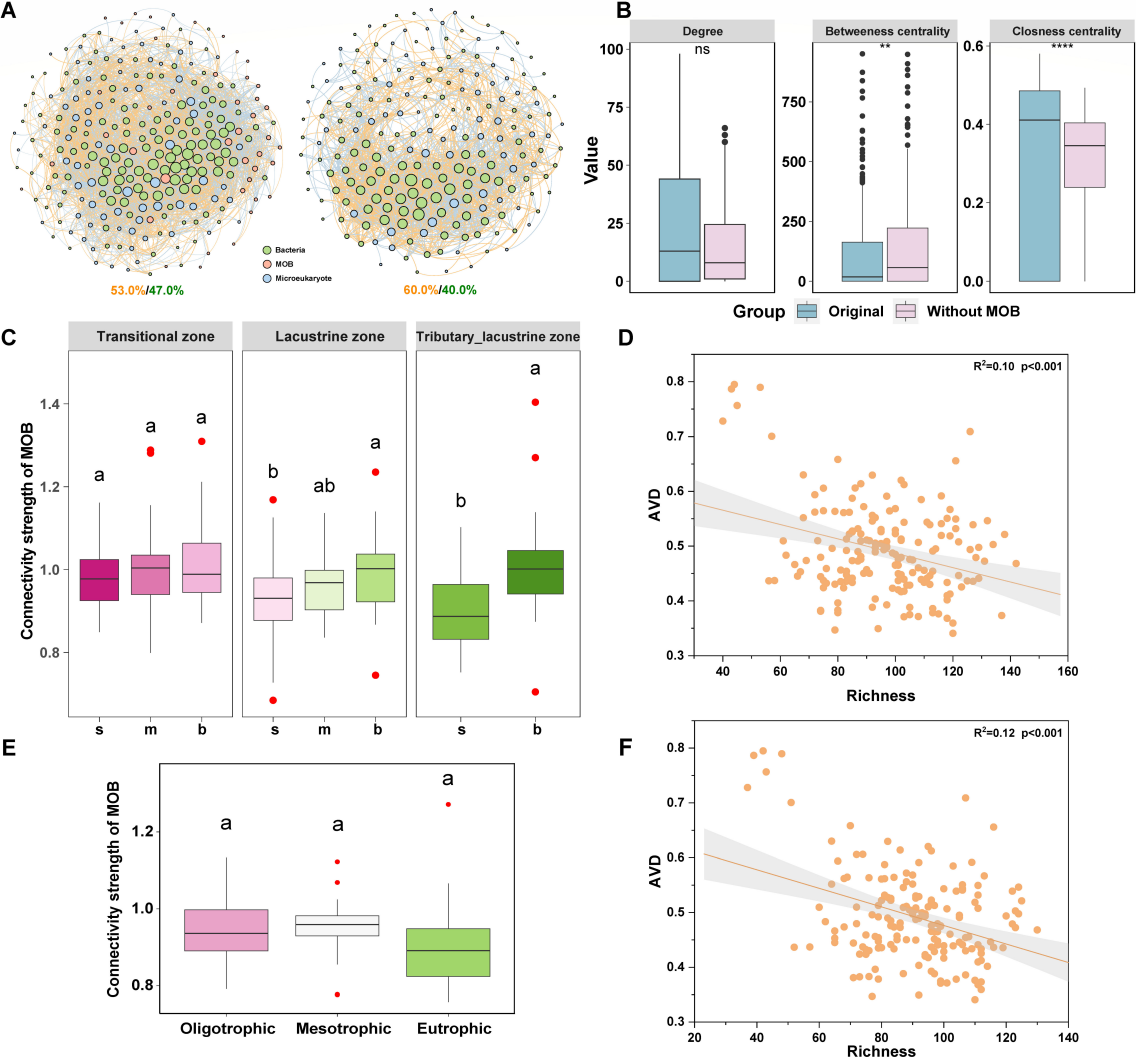
The relationship between biotic interactions of MOB and community stability across the hydrological and trophic gradient. (**A**) Co-occurrence patterns of bacteria, MOB, and microeukaryotes (left); bacteria, microeukaryotes (right) network in the riverine zone; Orange and green numbers in the graph indicated positive and negative correlation, respectively. (**B**) The boxplot of co-occurrence network topological parameters (including degree, betweenness centrality, and closeness centrality) of original (bacteria [including MOB] and microeukaryotes) (blue) and without MOB (pink) was shown in the riverine zone. Characteristics of other zones are shown in Fig. S3. Boxplots display median values (central line), interquartile ranges (boxes), 1.5× interquartile ranges and outliers (solid circles). Asterisks above boxes indicate statistically significant differences between groups based on Wilcoxon rank-sum tests: *P < 0.05, **P < 0.01, ***P < 0.001. Comparisons without asterisks denote non-significant differences (ns, *P >* 0.05). (**C**) Connectivity strength of MOB interactions with algae, zooplankton along the vertical gradient (surface [s], middle [m], and bottom [b] layers) within the transitional (T), lacustrine (L), and tributary-lacustrine (TL) zones. (**D**) The relationship between the richness of species (species interacted with MOB, including MOB) and the AVD index of microbial communities along the hydrological gradient. (**E**) Connectivity strength of MOB interaction with algae, zooplankton in the oligotrophic, mesotrophic, and eutrophic states, respectively. In the boxplots of panels **C and E**, hinges indicate the 25th, 50th, and 75th percentiles, whiskers indicate 1.5×  interquartile ranges, and outliers (solid circles). Lowercase letters above the boxes represent the results of the statistical significance test (*P* < 0.05, ANOVA with post hoc Tukey HSD test) for each group. (**F**) The relationship between the richness of species (species interacted with MOB, including MOB) and the AVD index of microbial communities along the trophic gradient.

To investigate the ecological role of MOB in microbial food loops, we established co-occurrence networks that included MOB and networks from which MOB was excluded (40) (Fig. 4A). Notable differences were observed in the topology of the co-occurrence network compared to the presence and absence of MOB in the riverine zone (Fig. 4B), suggesting that the role of MOB in the microbial community cannot be ignored. Furthermore, the proportion of interaction between MOB and algae was higher in the middle and bottom layers of the transitional, lacustrine, and tributary-lacustrine zones than in the surface layer (Fig. S4). However, the interaction of MOB-zooplankton was the greatest in the surface layer of the water column, especially in the riverine zone. The average proportion of interaction between MOB and zooplankton in the riverine zone (57.8%) and lacustrine zone (62.8%) was greater than the transitional zone (53.0%) and tributary-lacustrine zone (40.9%). Additionally, we observed that the connection strength of MOB with algae and zooplankton intensified as the water depth increased in the lacustrine zone and tributary-lacustrine zone (Fig. 4C). This pattern corresponded with the distribution of methane-derived carbon, underscoring the pivotal role of methane-derived carbon as a driving force in the productivity of the river-reservoir system. Furthermore, our analysis revealed a significant negative correlation between the richness of species interacting with MOB and the AVD index (Fig. 4D), confirming that MOB-mediated microbial communities may sustain community stability.

The interaction ratio of MOB-zooplankton was the highest in the oligotrophic (67.3%) and mesotrophic states (56.6%) (Fig. S5). And the proportion of MOB-algae interaction increased slightly with the increasing trophic status. In addition, the strength of MOB connections with algae and zooplankton was marginally higher in oligotrophic (0.95 ± 0.02) and mesotrophic (0.95 ± 0.02) systems than in eutrophic conditions (0.91 ± 0.03), though these differences lacked statistical significance (*P* > 0.05; Fig. 4E). Interestingly, we observed a significant negative relationship when we aligned the richness index of species that interacted with MOB against the AVD index (Fig. 4F). Natural connectivity, an indicator of network robustness to node removal, was higher in oligotrophic (41.4) and mesotrophic (38.6) systems than in eutrophic conditions (37.4) (Fig. S6). It is clear from both hydrological and trophic gradients that the biological interactions of MOB, especially MOB-zooplankton, may contribute to enhancing community stability.

### Community stability contributes to aerobic methanotrophy

**Fig 5:**
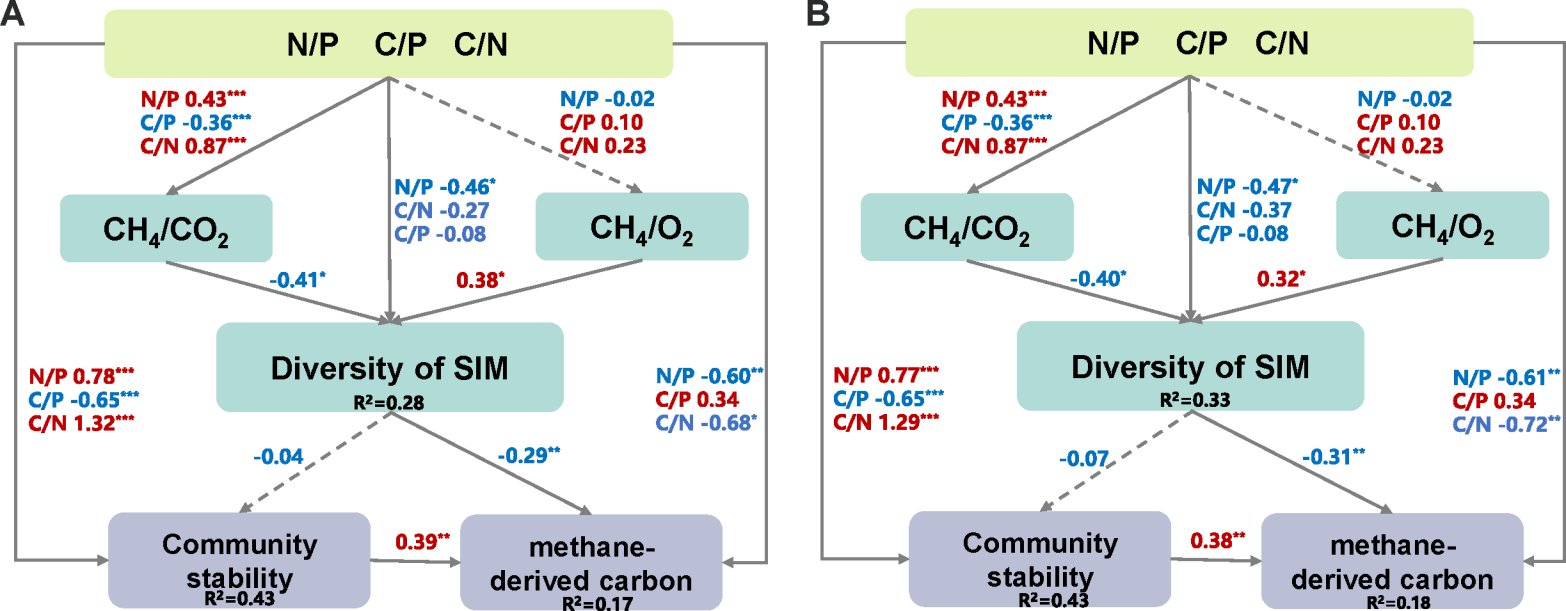
SEM describes the direct and indirect effects of nutrients (N/P, C/P, C/N), CH_4_/CO_2_, CH_4_/O_2_, and diversity of species that interacted with MOB (diversity of SIM) on community stability and methane-derived carbon in the hydrological gradient (**A**) and trophic gradient (**B**). The number adjacent to the arrow represents the effect size of the relationship. The red and blue numbers indicate positive and negative relationships, respectively; solid lines indicate significant relationships; dashed lines indicate non-significant relationships (*P* > 0.05); adjusted *P*-values are indicated by asterisks: *P < 0.05, **P < 0.01, ***P < 0.001. *R*^2^ denotes the proportion of variance explained for community stability and methane-derived carbon; model A, *P* = 0.131, Fisher’s *C* = 12.473, while model B, *P* = 0.15, Fisher’s *C* = 12.03. This indicated that the constructed model has an acceptable goodness of fit. N/P indicates the ratio of TN to TP; C/P indicates the ratio of DOC to TP; C/N indicates the ratio of DOC to TN.

The analysis of MOB interaction with microeukaryotes alongside environmental factors revealed that pH, WT, nutrients, and organic carbon are significant factors of species interactions (Fig. S7 and S8). Then, we constructed SEM to explore the influence of methane-derived carbon on small communities interacting with MOB as well as on the whole microbial community (Fig. 5). The results showed that the diversity of species interacting with MOB (diversity of SIM), community stability, and nutrients (N/P, C/P, C/N) merged as the most significant drivers for the production of methane-derived carbon (*R*^2^ = 17%, 18%). This suggests that methane-derived carbon is crucial for bacterial productivity within river-reservoir systems. Notably, community stability was found to be one of the important drivers of methane-derived carbon, highlighting that community stability may favor MOB growth and aerobic methanotrophy. On the other hand, the diversity of SIM and nutrients (N/P, C/P, C/N) was directly or indirectly impacted by the community stability (*R*^2^ = 43%), but not statistically significant for the former, unlike the above (Fig. 4D and F). This may be attributed to the complex interplay of multiple variables that may have masked the significant relationships.

### Microbial mechanisms for supporting community stability

**Fig 6:**
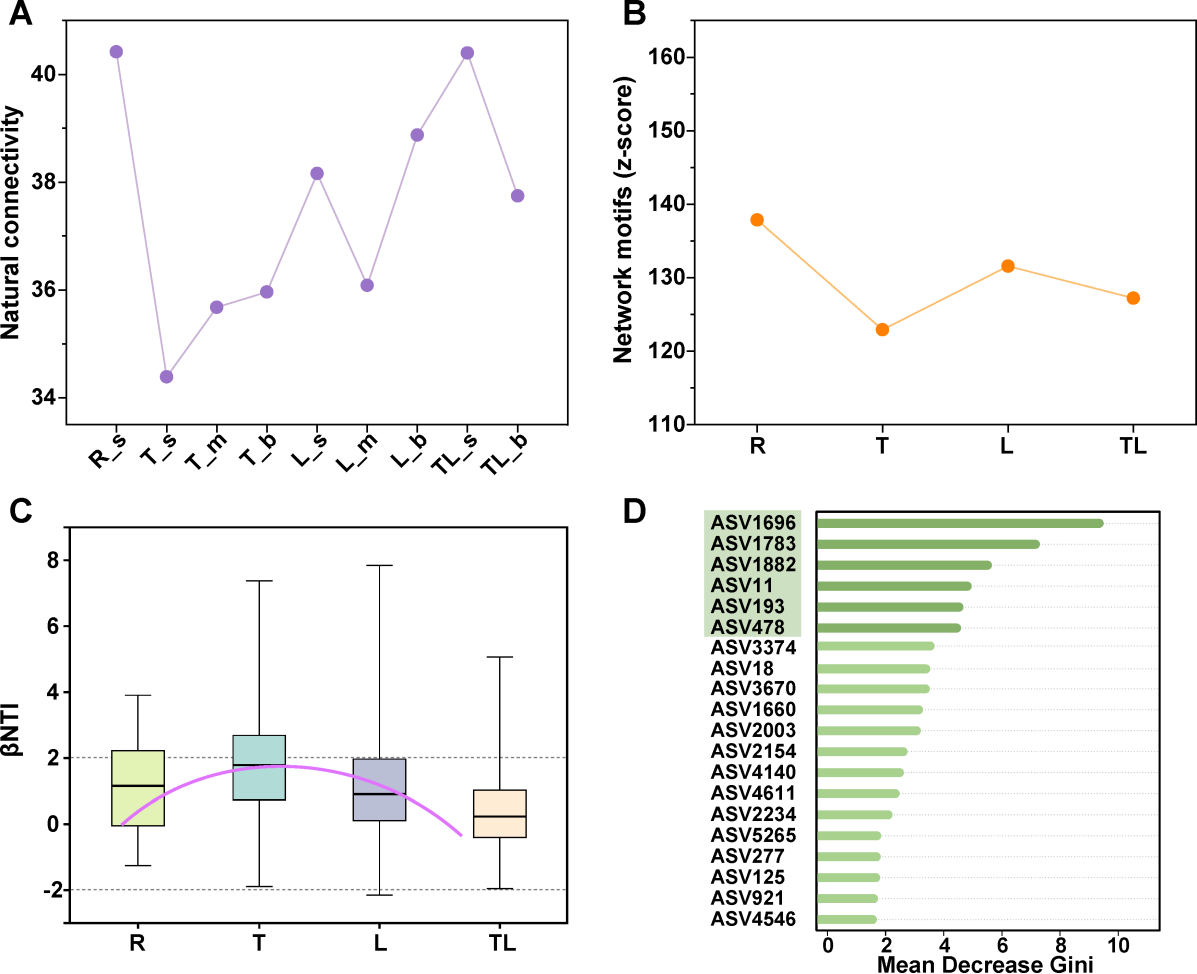
Community stability analysis and its microbial mechanisms. (**A**) Natural connectivity of microbial network in the surface (s), middle (m), and bottom (b) of the riverine zone (R), transitional zone (T), lacustrine zone (L), and tributary-lacustrine zone (TL). (**B**) Network stability is represented by calculating the z-score of the network motif (an overrepresentation of the empirical motif frequency compared to the motif frequency in a random network) along a longitudinal scale (without vertical stratification). (**C**) Different patterns of βNTI for bacterial communities in these four zones were analyzed. (**D**) Random forest model (ntree = 1,000) was trained on MOB species composition, yielding an out-of-bag (OOB) error rate of 17.98%. Key discriminant taxa (mean decrease Gini > 4), indicated by green shading, were strongly associated with hydrological gradients across zones. These species were identified as sensitive to damming.

Our findings indicated that the riverine zone has the greatest network stability (through natural connectivity), followed by the lacustrine zone, the tributary-lacustrine zone, and finally, the transitional zone (Fig. 6A). Longitudinal scale analysis showed that the riverine zone and lacustrine zone also have a higher overrepresentation of network motifs compared to the transitional zone (Fig. 6B). Both analytical approaches consistently identified riverine and lacustrine zones as exhibiting the highest community stability, demonstrating robust cross-scale stability patterns in these ecosystems.

Our analysis of bacterial and microeukaryotic community structures identified marked differences between the riverine zone and the lacustrine zone (Fig. S9). Yet, the community structure of the less stable transitional zone bears a closer resemblance to that of the lacustrine zone. Figure S10 revealed a significant hydrological gradient in community similarity, with the lacustrine zone showing the highest values (0.408 ± 0.003), followed by the riverine zone which exhibited greater similarity than the tributary-lacustrine zone (0.192 ± 0.004) but no significant difference from the transitional zone (0.378 ± 0.004), collectively suggesting enhanced community resilience in both riverine and lacustrine zones. Then, examining the βNTI across the riverine zone, transitional zone, and lacustrine zone on a longitudinal scale—excluding the tributary-lacustrine zone—revealed a concave downward parabolic trend (Fig. 6C; Fig. S11). This analysis revealed that deterministic processes (|βNTI| > 2) accounted for 43.7% of processes in the transitional zone, surpassing the proportions observed in the riverine (28.1%) and lacustrine zones (24.0%). Nevertheless, stochastic processes still predominated across all zones, with the transitional zone exhibiting a marginally higher percentage of deterministic processes compared to other regions. In the context of the niche breadth index, we observed a parabolic pattern on a longitudinal scale (Fig. S12). The highest position showed that communities in the lacustrine zone harbored more generalists with higher metabolic flexibility, while the lowest position in the riverine zone harbored more specialists. The transitional zone has an intermediate niche breadth index, which means that species in this region may be neither highly specialized nor fully generalized.

We further identified the sensitive MOB species to damming that exhibited significant variation in the hydrological gradient. The supervised random forest classifier identified ASV1783, ASV1882, ASV193, ASV478, ASV1696, and ASV11 as the sensitive species (Fig. 6D). The relative abundance of these sensitive MOB species varied significantly across zones (Fig. S13). Our study revealed a fascinating detail: in the riverine zone, the keystone species (characterized by high degrees and low betweenness centrality) was the Alpha-MOB led by ASV1783. Conversely, in the lacustrine zone, the keystone species was the Gamma-MOB dominated by ASV11. These phenomena indicated that the core MOB species that maintain community stability are distinct.

## DISCUSSION

The intricate interplay between aerobic methanotrophy and microbial community stability can help elucidate critical ecological dynamics behind the biogeochemical process of CH_4_ in freshwaters. Through a multidimensional approach that considers both the hydrological and trophic gradients, we seek to deepen the understanding of the function of aerobic methanotrophy in microbial food loops, providing a holistic understanding of the role of MOB and methane-derived carbon in freshwaters.

### Methane-derived carbon

MOB can provide organic carbon to heterotrophic bacteria or zooplankton, while benefiting from the nutrients they produce (41, 42). Extensive studies have shown that methane-derived carbon serves as an essential carbon source through the predation of MOB by zooplankton (15, 16, 43). Sanseverino et al. (43) showed that the assimilation of methane-derived carbon can be transferred as a carbon source in aquatic food webs, even reaching up to the fish level. Kankaala et al. (20) reported that methane-derived carbon contributes approximately 23%–81% to the zooplankton in stratified humic lakes. In large shallow lakes, methane-derived carbon can support roughly 20% and is consistently relatively stable in larval bodies (44). Yet, the role of methane-derived carbon in microbial food loops needs further elaboration along the hydrological gradients structured by the river-reservoir system.

In our research, higher methane-derived carbon contribution occurred at the bottom of the riverine zone (22.6%) and transitional zone (15.2%), which may be due to the accumulation of hotspot CH_4_ by sedimentation (34). The increase in methane-derived carbon with increasing water depth suggests that in deeper areas it may also reflect sediment anoxic conditions that favor methanogenesis and subsequent MOB activity (9). In the lacustrine zone, the contribution of methane-derived carbon is irrespective of water depth. The consistent methane-derived carbon contribution might be owing to the lack of thermal stratification like that found in natural lake systems, resulting in uniformity of conditions. The strong positive correlation observed between methane-derived carbon and community stability indicates that the availability of methane-derived carbon as an additional carbon source may alleviate competition among species, thereby enhancing species diversity, which in turn strengthens community stability (45). In the stable riverine zone, methane-derived carbon is abundant; however, the higher CH_4_ flux may be due to substrate availability rather than suppressed aerobic methanotrophy. The strength of biotic interaction among MOB may be fostered by their role as a carbon source. Conversely, in the stable lacustrine zone, although methane-derived carbon is less abundant, the high degree of niche differentiation among species may enhance aerobic methanotrophy, resulting in lower CH_4_ flux (0.05 mmol·m^−2^·day^−1^). Here, the strength of biotic interactions among MOB is not only due to cooperation arising from their carbon supply but may also be facilitated by interactions with other species that promote aerobic methanotrophy. Thus, stable communities with higher interaction strength have a positive effect on the substance metabolism for specific functions (46).

There is a non-linear trend between methane-derived carbon contribution and trophic states. It suggests that the trophic gradient, typically associated with productivity, does not uniformly influence the role of CH_4_ as a carbon source. The stable oligotrophic state community exhibits a lower contribution of methane-derived carbon at 10.8%, yet the enhanced strength of biotic interactions among MOB is associated with reduced CH_4_ emissions of 0.05 mmol·m^−2^·day^−1^, likely due to the effect of stronger aerobic methanotrophy. In mesotrophic communities, the contribution of methane-derived carbon was the highest (17.5%), along with the most substantial biotic interactions among MOB, leading to lower CH_4_ emissions (0.12 mmol·m^−2^·day^−1^). This reinforces the notion that solid MOB interactions are more conducive to aerobic methanotrophy in a stable community.

### Community stability

Community stability is influenced by an interplay of species diversity and the interactions among different species, as demonstrated by May (47). This suggests that both the variety within the ecological community and the complex relationships between its members are critical factors that contribute to community-level resilience and equilibrium. Community stability will essentially increase with species diversity due to the statistical averaging of fluctuations in the abundance of different species (48). When community stability changes, it can impact the relative abundance and diversity of species, subsequently influencing their metabolic activities and ecological functions.

Along the hydrological gradient, species diversity was notably higher in the riverine zone (Fig. S14), where community stability was the greatest, compared to the other zones (Fig. 6A). This pattern is also consistent across the trophic gradient, that is, the oligotrophic and mesotrophic states with higher species diversity also have greater community stability. Increased species diversity can enhance community stability by promoting compensatory dynamics among species (also known as species asynchrony), which helps to mitigate external disturbances (49). It is also probably attributed to eutrophication weakening the effect of diversity on stability (50). This pattern aligns with experiments in plant communities, where adding nutrients (e.g., nitrogen) similarly enhanced stability by increasing diversity (51).

The strength of biological interactions for MOB increased with increasing water depth in both stable and less stable communities. However, the increase in interaction strength was more pronounced in the stable lacustrine zone, ranging from 0.92 to 1.0. Furthermore, the identification of keystone MOB species sensitive to damming exhibited a shift from Alpha-MOB in the riverine zone to Gamma-MOB in the lacustrine zone. This transition may be attributed to the fact that Alpha-MOB possesses a smaller cell size, making it easier to adapt for more efficient nutrient uptake in the riverine zone (6). Conversely, the Gamma-MOB, with their broader metabolic capabilities, are better suited to the more stable and nutrient-rich conditions of the lacustrine zone, where they can thrive in a broader range of available resources. This indicated a disparity in the adaptive capabilities and plasticity among various MOB species. In our results, a stable microbial community was also characterized by intensified species interactions between MOB and zooplankton. Co-occurrence network analyses further showed that the main MOB species interacting with zooplankton succession from Alpha-MOB in the riverine zone to Gamma-MOB in the lacustrine zone, reconfirming the distribution and ecological roles of MOB in different habitats. By facilitating nutrient cycling, predation, and other ecological processes, MOB and their interactions can influence the overall structure and function of microbial communities, thereby promoting their stability and confirming that MOB is a stabilizing force for microbial communities (52). The mechanism may be attributed to the fact that CH_4_ oxidation by MOB produces biomass and metabolic by-products that provide nutrients for other microorganisms, thus fostering a more diverse and stable microbial community (12). This highlights the importance of considering the complex web of interactions within microbial communities when assessing their stability.

In addition, network motifs revealed that cross-domain interactions (e.g., MOB-microeukaryote) constituted 41.7%–49.3% of all microbial associations across the river-reservoir systems (Fig. S15A), with facilitation-driven motifs (cycle facilitation [cycfac], facilitation-mediated competition [facmcom]) overrepresenting and predominating (especially in the riverine, lacustrine, and tributary-lacustrine zones) (Fig. S15B and C), which probably promoted species coexistence and persistence (45). Notably, cycfac exhibited a positive (though non-significant) correlation with bacterial and microeukaryotic diversity (Fig. S15D), whereas an increase in facmcom was associated with a decrease in microbial diversity, indicating that network motifs could potentially enhance microbial diversity in river-reservoir systems through direct and indirect cooperation mechanisms. This also aligns with the observed dominance of positive associations in co-occurrence networks (Fig. S3A). Collectively, these findings position interspecies cooperation (e.g., cross-feeding of methane-derived carbon and nutrients) as a critical driver of microbial diversity and community stability in aquatic systems.

**Fig 7:**
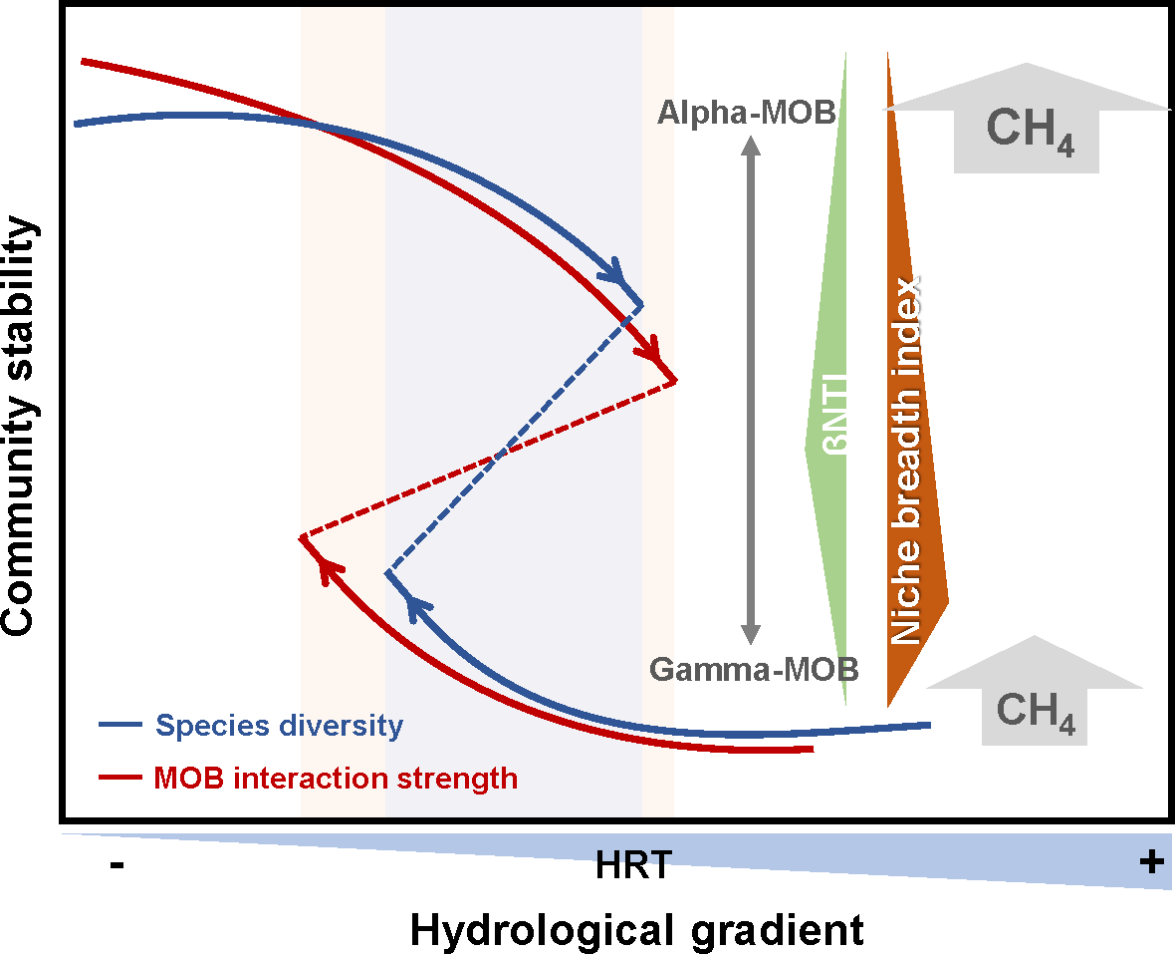
Characteristics of community stability in the riverine, transitional, and lacustrine zones along the hydrological gradient in river-reservoir systems; HRT, hydraulic residence time.

Thus, we encapsulate the features of community stability in the riverine, transitional, and lacustrine zones along the hydrological gradient (Fig. 7). Species diversity and strength of interaction with MOB are key elements in maintaining community stability, besides stochastic processes (fewer environmental factor influences) or higher niche differentiation also contributing to community stability. We have found that the stable riverine zone is influenced only by environmental factors such as pH, WT, and NO_2_-N. In the less stable transitional zone, physicochemical indicators, such as SPS, POC, DOC, and nutrients (N/P, C/P, C/N), all impact species diversity. In previous studies, it has been reported that SPS is an important factor influencing species diversity (53, 54). The parabolic trend of βNTI and niche breadth index across the hydrological gradient suggests a shift from less stochastic processes in the transitional zone to more stochastic processes in the riverine and lacustrine zones. This distinction in ecological processes further illustrates the varying impacts on community stability, which cause species to adapt frequently to changes in the environment, potentially resulting in them exhibiting lower community stability in the transitional zone (55). Chen et al. (56) discovered that ecotone habitats between desert and oasis exhibit the simplest network structure. This could be attributed to the regulation of community stability through the influence of total N, P, and OC on microbial interactions. Additionally, microbial communities in the lacustrine zone with a high niche breadth index (i.e., species adaptable to diverse conditions) provide greater metabolic flexibility, which is critical for maintaining community stability (Fig. S12). Microbial communities in oligotrophic and mesotrophic states exhibit higher niche breadth index and are predominantly governed by stochastic processes (33). This pattern suggests that their stability may be enhanced through species metabolic flexibility, random turnover, and compensatory dynamics where functionally overlapping taxa buffer perturbations by substituting for one another during environmental changes.

Our findings establish aerobic methanotrophy as the critical process governing methane-derived carbon dynamics in river-reservoir systems. MOB mediate CH_4_ oxidation to CO_2_ and biomass, reducing emissions while channeling carbon into microbial food webs via cross-domain interactions where organic carbon from MOB fuels heterotrophs in exchange for growth-enhancing metabolites. Critically, methane-derived carbon, acting as a carbon subsidy in ecosystems, is regulated by community stability, which arises from the interplay of species diversity, interaction strength, niche differentiation, and ecological processes, collectively maintaining functional persistence under environmental fluctuations. The greater contribution of methane-derived carbon in the riverine zone and mesotrophic states reflects how physical habitat heterogeneity (e.g., hydrological conditions) governs CH_4_ concentration and accessibility, while nutrient availability modulates the efficiency of aerobic methanotrophy. This interplay highlights strategies for managing aquatic systems to optimize aerobic methanotrophy while sustaining microbial food web productivity. However, if consumers become too dependent on methane-derived carbon, this could reduce the functioning of aerobic methanotrophy and affect the nutritional quality of consumers due to their lack of polyunsaturated fatty acids (57), which may have a greater impact on community stability.

These findings position aerobic methanotrophy as the critical process connecting methane-derived carbon dynamics to broader ecosystem functions, providing a framework for modeling CH_4_ flux in aquatic systems. Although our research takes a methane-derived carbon perspective and emphasizes its relationship with CH_4_ concentration and community stability, future studies need to incorporate *in situ* monitoring of aerobic methane oxidation rates in order to gain a deeper understanding of these coupled processes.

## Data Availability

All sequencing data have been deposited at the National Center for Biotechnology Information (NCBI) Sequence Read Archive (SRA; available at http://www.ncbi.nlm.nih.gov/sra/) under the accession numbers PRJNA827670, PRJNA738555, and PRJNA1251314.

## SUPPLEMENTAL MATERIAL

Supplemental material msystems.00530-25-s0001.pdf Supplemental methods, Figures S1-S15, and Tables S1 and S2.

ASM does not own the copyrights to Supplemental Material that may be linked to, or accessed through, an article. The authors have granted ASM a non-exclusive, world-wide license to publish the Supplemental Material files. Please contact the corresponding author directly for reuse.
