# Supplementary material for: Stable microbial community promotes aerobic methanotrophy in a large river-reservoir system
Source: mSystems. 2025 Jun 25;10(7):e00530-25. doi: 10.1128/msystems.00530-25 (PMC12282086; doi:10.1128/msystems.00530-25)
Supplement: Supplemental material — Supplemental methods, Figures S1-S15, and Tables S1 and S2. [file msystems.00530-25-s0001.pdf]

Supplementary Materials

Title: Stable microbial community promotes aerobic methanotrophy in a large river-reservoir system

Authors: Qiong Tang[1,2,3], Zhe Li[2,3*], Lunhui Lu[2,3], Yan Xiao[2,3], Xinghua Wu[4], Dianchang Wang[4]

1 Chongqing Jiaotong University, Chongqing 400074, China.

2 CAS Key Lab of Reservoir Environment, Chongqing Institute of Green and Intelligent Technology, Chinese Academy of Sciences, 400714, Chongqing, China.

3 College of Resources and Environment, Chongqing School, University of Chinese Academy of Sciences, 400714, Chongqing, China.

4 National Engineering Research Center of Eco-Environment in the Yangtze River Economic Belt, China Three Gorges Corporation, Wuhan, 430010.

[*] Corresponding author: lizhe@cigit.ac.cn

Supplementary of Methods

Estimate the relative contributions of methane-derived carbon

It was hypothesized that the measured DOC concentration in the water column is a mixture of methane-derived carbon and DOC formed by the degradation of POC through biochemical processes.

Three reasons can support this hypothesis: 1) The majority of organic carbon in aquatic ecosystems is found in its dissolved form [1]; 2) Pohlman et al. demonstrated that methane-derived carbon constitutes a significant byproduct of microbial CH_4 consumption [2]; 3) In natural waters, POC can be converted to DOC through biodegradation, photochemical degradation, and physical processes [3]. Here, the concentration of DOC in the upper Yangtze was higher than that of POC, and their $\delta^{13}C$ isotopic fractionation factors were very close (Table S2), which may indicate that the transformation process may be one of the primary sources of the increase in the DOC concentration.

Therefore, a two-source mixing model was employed to determine the relative contribution of methane-derived carbon and POC in the DOC [4, 5]. The calculation was based on the following equation:

$$\%\text{methane-derived carbon in DOC} = \frac{\delta^{13}\text{C\_DOC} - \delta^{13}\text{C\_POC}}{\delta^{13}\text{C\_CH}_4 - \delta^{13}\text{C\_POC}} \quad (1)$$

where $\delta^{13}C\_DOC$ is the measured $\delta^{13}C$ value of the DOC, $\delta^{13}C\_CH_4$ is the average value of CH_4 in all the sampling sites, $\delta^{13}C\_POC$ is the measured $\delta^{13}C$ value of the POC. This calculation does not consider carbon isotope fractionation by aerobic methanotrophs [5, 6].

Network analysis

Microbial co-occurrence network analysis is a common approach to investigate the interaction relationship between microbial species [7, 8]. To explore the microbial interaction patterns between bacteria and microeukaryotes (protozoan, metazoan, algae), we selected species with a relative abundance greater than 0.01% in any sample for network construction [9]. Protozoan and metazoan are hereafter collectively referred to as zooplankton. The merged tables (bacteria and microeukaryotes ASV tables) served

as the input for SparCC analysis. This analysis employed compositionality-robust correlations based on the median of the 20 iterations and estimated pseudo p-values from 100 bootstrap samples (two-sided) [10]. Subsequently, all possible pairwise correlations (r) among these ASVs were calculated. Only those correlations that were statistically significant and robust ($p < 0.05$, $|r| > 0.3$) were deemed valid for further analysis [11]. The SpiecEasi package in R (version 4.1.1) was utilized to perform sparse correlations for calculation purposes. All topological parameters (e.g. degree, betweenness centrality and closeness centrality) analyses and network visualizations were conducted using the igraph package in R and Gephi (version 0.9.2).

Network motifs

To enhance our understanding of microbial ecological network structure, we assessed network module prevalence across three phylotypes, commonly known as network motifs [12]. Network motif is the basic building block of network architecture, capturing the interactions among triplets of species [12]. These association modules construct complex aquatic microbial communities. Due to undirected associations, there are seven types of aquatic ecological networks as follows [13, 14]: 1) cycle facilitation, cycfac; 2) cycle competition, cyccom; 3) facilitation-mediated competition, facmcom; 4) competition-mediated facilitation, commfac; 5) transitive facilitation, tranfac; 6) transitive competition, trancom; and 7) transitive competition and facilitation, trancomfac. Thus, we employed network motifs encompassing multiple associations among species to investigate how complex biotic interactions contribute to promoting microbial diversity.

Community Statistics

Community stability analysis

Community stability was calculated in three approaches. Firstly, we analyze community stability using the results of the network motif described above. When evaluating motifs in isolation from their encompassing larger network, the persistence of these motifs serves as a reliable probabilistic metric for assessing the stability of the whole network [15]. Therefore, to estimate the overrepresentation of network motifs relative to random networks, we performed z-score calculations on 999 random

networks, which also served as an indicator of network stability [13, 15].

Secondly, we employed the average variation degree (AVD) index to assess the stability of the microbial community [16]. This metric was determined by calculating the deviation of the relative abundance of ASVs from the mean of the normal distribution across various samples. A lower AVD value indicates greater stability within the microbial community. The equation is as follows:

$$\mathrm{AVD} = \frac{\sum_{i=1}^{n} |a_i|}{\mathrm{k} \times \mathrm{n}} \quad (2)$$

$$|a\mathrm{i}| = \frac{|x_i - \bar{x}_i|}{\delta_i} \quad (3)$$

where k is the number of samples and n is the number of ASVs. a_i represents the variability of $\mathrm{ASV_i}$, x_i is the abundance of $\mathrm{ASV_i}$ in each sample, and $\bar{x}_\mathrm{i}$ and δ_i are the mean and standard deviation of the abundance of $\mathrm{ASV_i}$ in all samples, respectively.

The third approach is to consider network stability by computing the natural connectivity of a complex network, which is widely used to reveal the robustness of microbial networks [17, 18].

Connectivity strength analysis

We quantified the strength of connectivity among MOB, algae and zooplankton using the cohesion method to calculate the ratio of the absolute value of negative to positive connectivity [19]. Cohesion can provide insight into associations between taxa caused by positive and negative interactions, as well as associations caused by similarities and differences in the ecological niches of microbial taxa. For each sample, two cohesion values were calculated as the sum of the weights and taxon abundance of significant positive or negative correlations between taxa. Negative and positive connectivity values range from -1 to 0 and 0 to 1, respectively. The ratio of their absolute values is used to characterize the relative strength.

Community assembly processes

To assess the relative importance of deterministic and stochastic in microbial community assembly, we calculated the beta nearest taxon index (βNTI) using a null model (999 randomizations) [20]. In this method, we employed the beta mean nearest taxon distance (βMNTD) metric to quantify the pairwise phylogenetic turnover

between communities. This measure reflects the degree of changes in species composition and phylogenetic relationships across community pairs. Additionally, we utilized the βNTI to assess the environmental impact on these communities. The βNTI is derived from the standard deviation between the observed βMNTD and the βMNTD values obtained from a null model, which simulates random community assembly processes. A significant deviation from the null model's βMNTD values indicates a strong environmental influence on the phylogenetic structure of the microbial communities. $|\beta NTI| > 2$ represents deterministic processes, whereas $|\beta NTI| < 2$ represents stochastic processes.

Niche breadth index

To explore the bacterial and microeukaryotic communities along a hydrological gradient, we employed the Levins' niche breadth index to quantify the metabolic flexibility of these microbial groups [21, 22]. This approach allows for a deeper understanding of the ecological strategies and resilience of microbial communities in response to changes in environmental factors.

Random forest identification

We utilized the random forest (RF) algorithm to pinpoint MOB species sensitive to hydrologic perturbations. The mean decrease Gini (MDG) was employed to evaluate the significance of the variables, with higher MDG values indicating greater importance of the corresponding features. We constructed one hundred RF replicates, each comprising 1000 trees, using the R package randomForest to establish a robust ranking of feature importance [23]. Subsequently, we identified the species most responsive to hydrologic perturbations by determining the optimal breakpoints. This was achieved using the linear regression model and the breakpoints function available in the R package strucchange [24].

Data compilation and analysis strategy

The datasets from field sampling campaigns were divided into four groups. Initially, sampling sites were categorized into three subsets: riverine zone, transitional zone, and lacustrine zone in the main stem of the Yangtze. Additionally, the fourth subset is the tributary-lacustrine zone, including the GY sampling site in the TGR and sampling sites

in the SZT. This zone is characterized by a longer hydraulic retention time and a shallower water column compared to the main stem. For brevity, we'll use the abbreviations R, T, L, and TL to represent the riverine zone, transitional zone, lacustrine zone, and tributary-lacustrine zone, respectively. Another dimension is the trophic gradient, where we determined the oligotrophic, mesotrophic, and eutrophic states based on the trophic level index at each sampling site, as described in our previous publication [26].

In this study, we chose to utilize a trophic gradient perspective to underpin our analysis of the hydrological gradient, which provided a multidimensional view. However, it is worth noting that hydrological gradients and trophic gradients are not entirely independent of each other in the river-reservoir system. This may limit our current data analysis and explanation.

Supplementary Figures

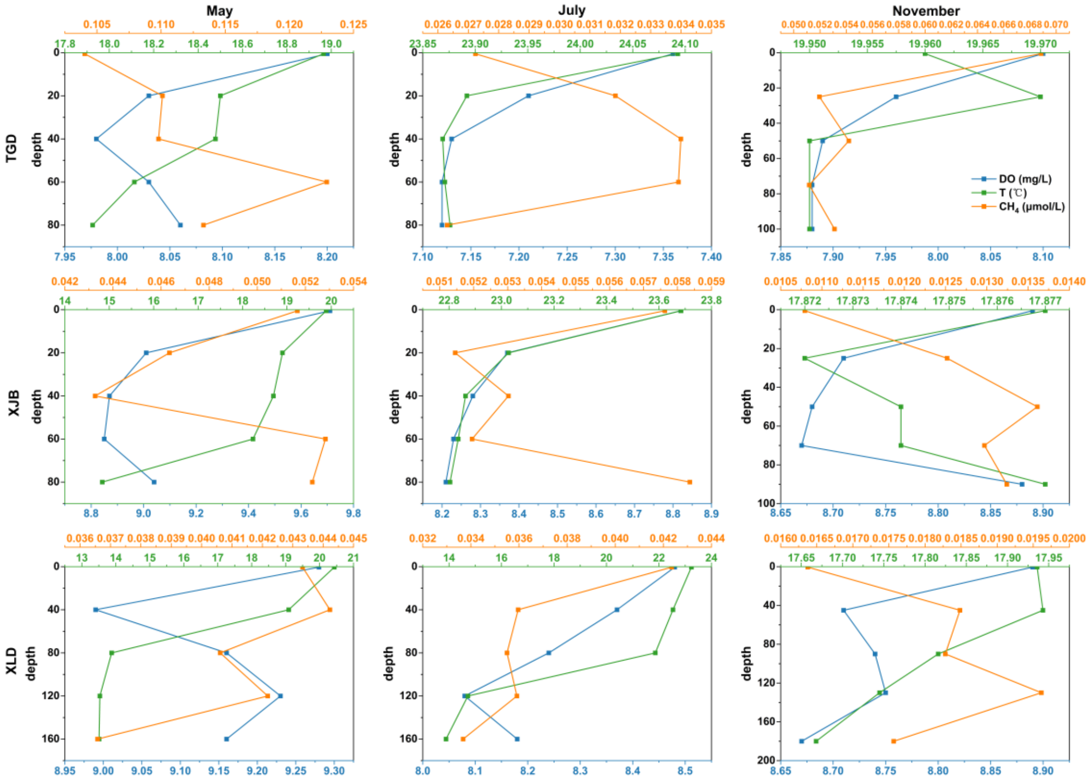

Figure S1. The vertical profiles of pre-dam of dissolved oxygen (DO), water temperature (T), and CH_4 concentration during May, July, and November in the Three Gorges Dam (TGD), Xiangjiaba (XJB), and Xiluodu (XLD), indicated by blue, green and orange color lines, respectively.

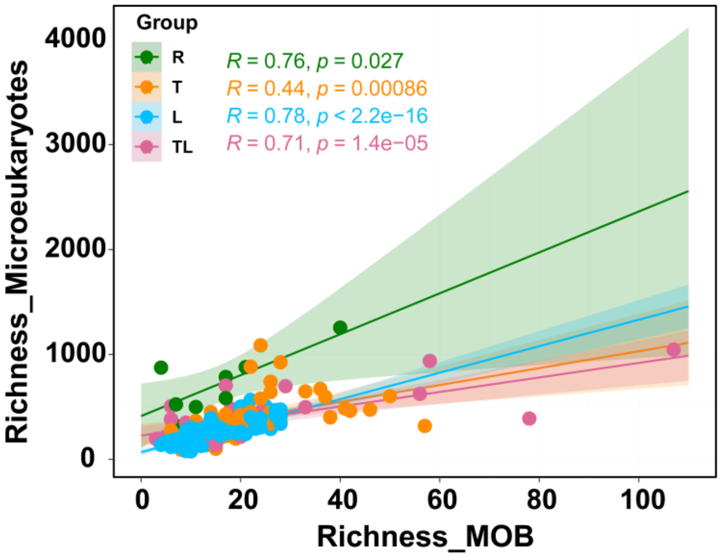

Figure S2. Relationship between richness index of MOB and microeukaryotes in the four zones; Green, orange, blue, and purple color represent the riverine zone (R), transitional zone (T), lacustrine zone (L), and tributary-lacustrine zone (TL), respectively.

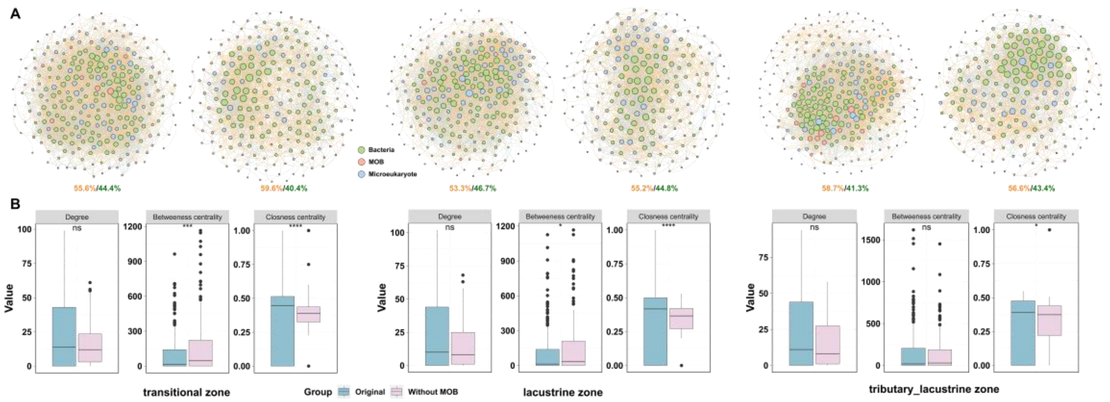

Figure S3. A Co-occurrence patterns of bacteria, MOB and microeukaryotes (sub-figure left), bacteria, microeukaryotes (sub-figure right) network in the transitional zone, lacustrine zone and tributary_lacustrine zone, respectively. Orange numbers in the graph indicated positive correlations and green numbers showed negative correlations. **B** The boxplot of co-occurrence network topological parameters (including degree, betweenness centrality, and closeness centrality) of original (bacteria [including MOB] and microeukaryotes) (blue) and without MOB (pink) was showed in the transitional zone, lacustrine zone, and tributary_lacustrine zone.

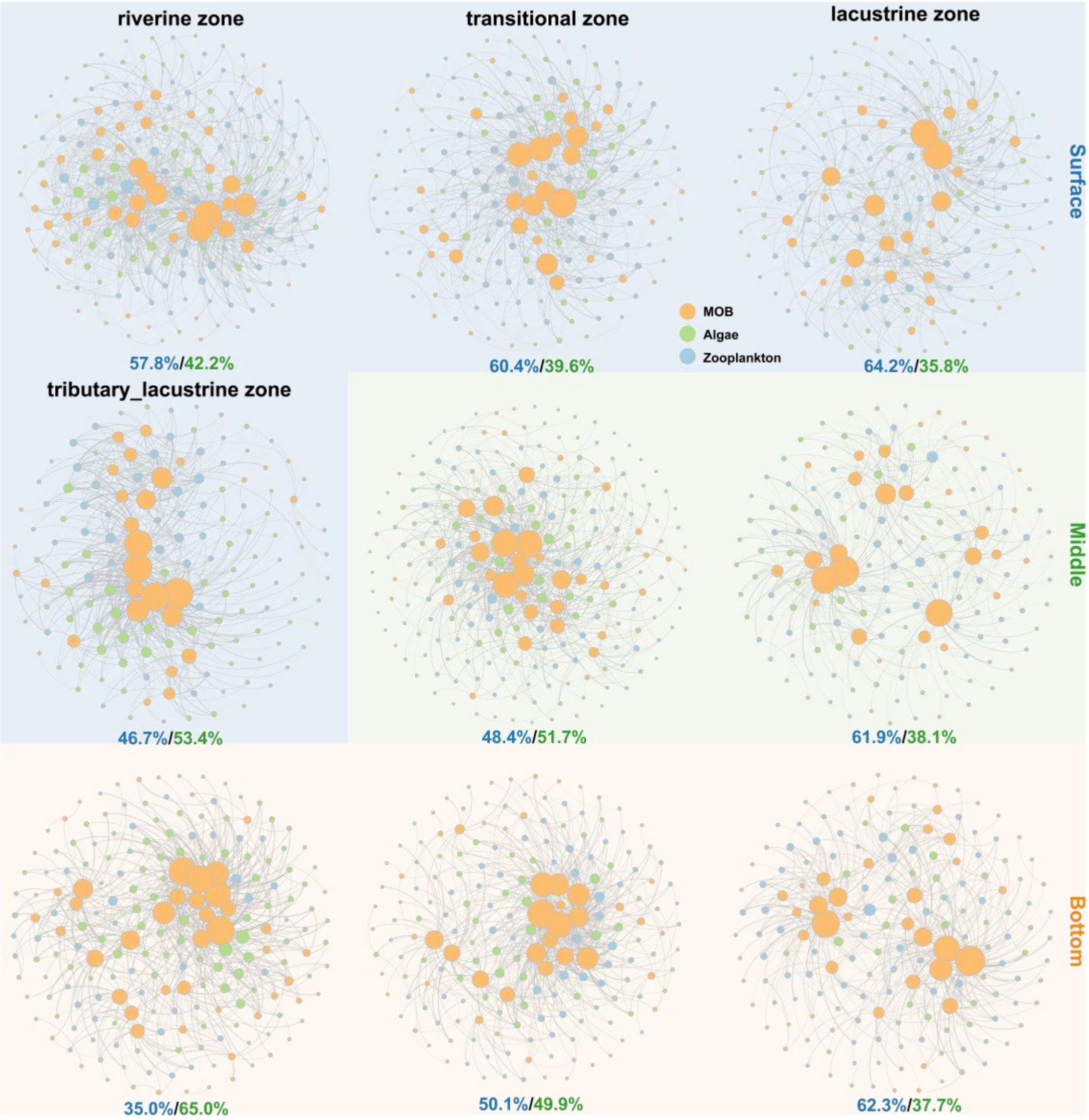

Figure S4. Interaction relationships of sub-network between MOB-algae and MOB-zooplankton vertically from the riverine zone, transitional zone, lacustrine zone and tributary_lacustrine zone; blue shading area: surface layer; green shading area: middle layer; orange shading area: bottom layer; blue numbers below the sub-network plots: MOB-zooplankton interactions ratio; green numbers: MOB-algae interactions ratio.

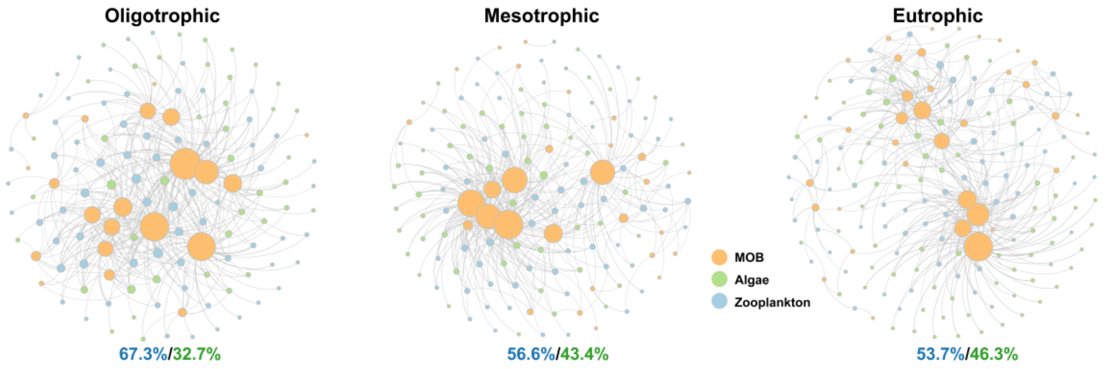

Figure S5. Interaction relationships of sub-network between MOB-algae and MOB-zooplankton in the oligotrophic, mesotrophic, and eutrophic states, respectively; blue numbers below the sub-network plots: MOB-zooplankton interactions ratio; green numbers: MOB-algae interactions ratio.

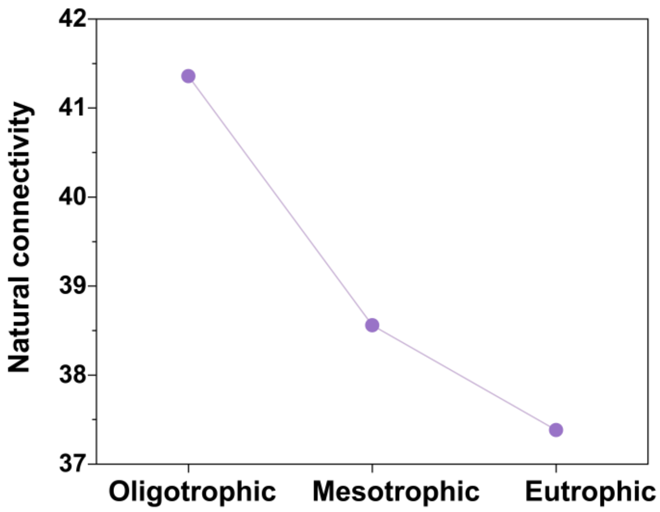

Figure S6. Natural connectivity was showed in the oligotrophic, mesotrophic and eutrophic states, respectively.

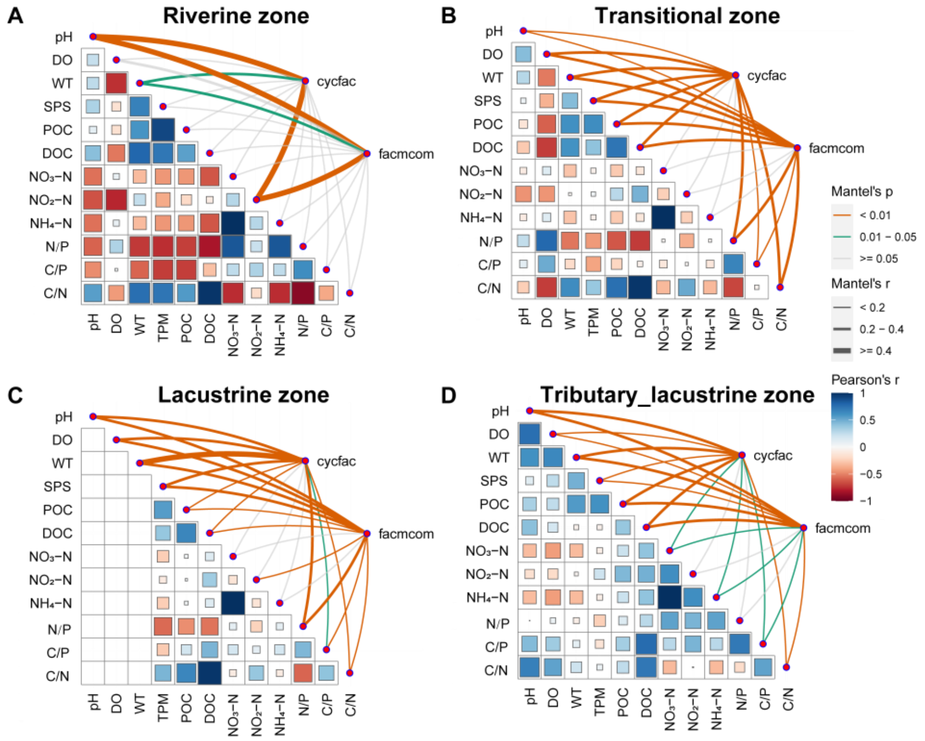

Figure S7. Correlation of overrepresented motifs (cycfac, facmcom) of MOB interacted with microeukaryotes with environmental factors in the riverine zone (A), transitional zone (B), lacustrine zone (C) and tributary_lacustrine zone (D). N/P indicates the ratio of TN to TP; C/P indicates the ratio of DOC to TP; C/N indicates the ratio of DOC to TN.

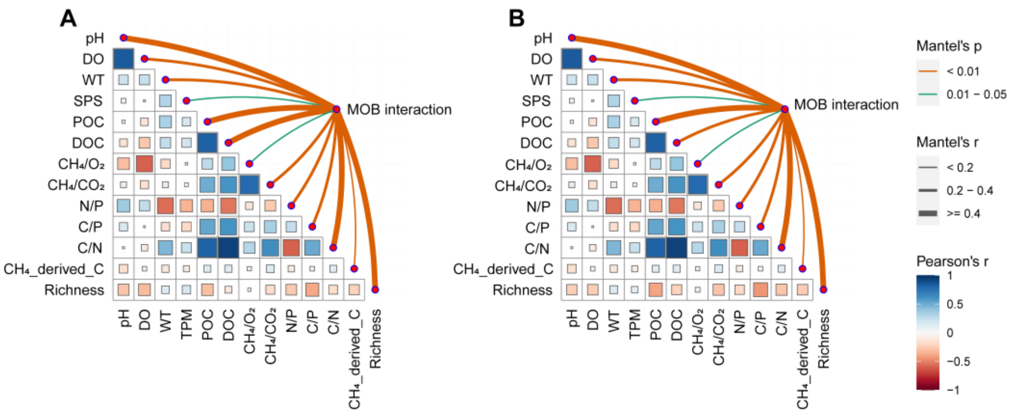

Figure S8. Correlations of species interacted with MOB with environmental factors in the hydrological gradient (A) and trophic gradient (B); N/P indicates the ratio of TN to TP; C/P indicates the ratio of DOC to TP; C/N indicates the ratio of DOC to TN; CH_4_derived_C represents methane derived carbon.

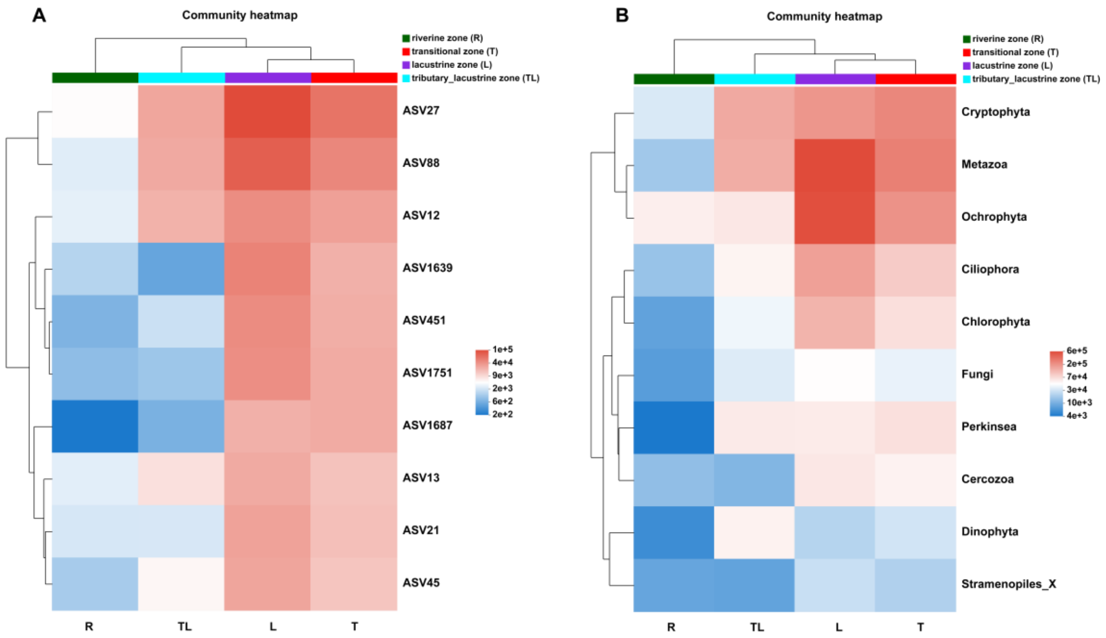

Figure S9. Heatmap plots are used to present information on bacterial (A), microeukaryotic (B) community composition in terms of a color gradient and clustering based on similarity in abundance between species and samples.

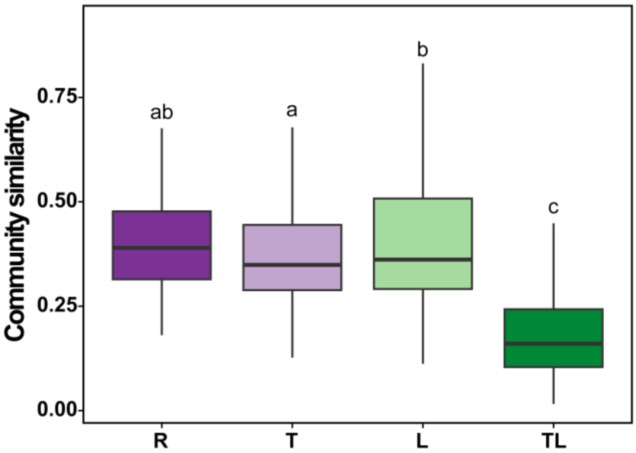

Figure S10. Community similarity (Bray-Curtis similarity) among the riverine zone (R), transitional zone (T), lacustrine zone (L), and tributary-lacustrine zone (TL) of the microbial community at the ASV level. Lowercase letters above the boxes represent the results of statistical significance test ($P < 0.05$, ANOVA with post-hoc Tukey HSD Test) for each group. Groups with different letters indicate significant differences between groups, and groups with the same letter means non-significant differences between groups.

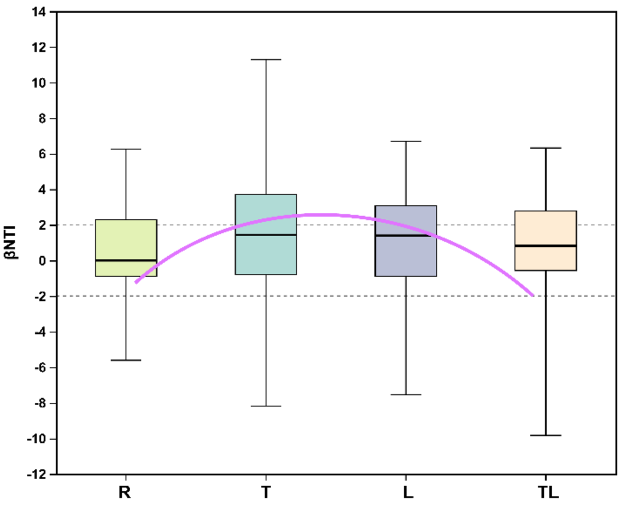

Figure S11. Different patterns of βNTI for microeukaryotic communities in the riverine zone (R), transitional zone (T), lacustrine zone (L) and tributary-lacustrine zone (TL) were analyzed.

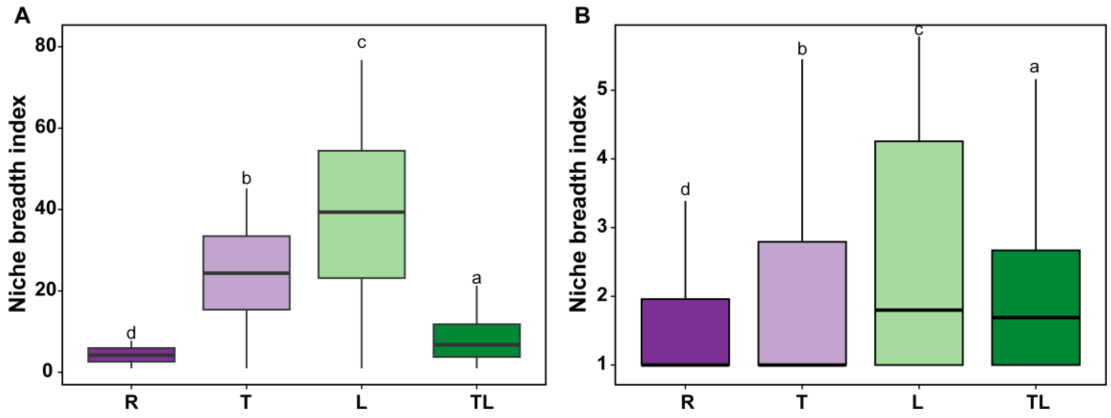

Figure S12. Comparison of niche breadth index for bacteria (A), microeukaryotes (B) in the riverine zone (R), transitional zone (T), lacustrine zone (L) and tributary-lacustrine zone (TL). Lowercase letters (a, b, c, d) above the boxes represent the results of statistical significance test ($P < 0.05$, ANOVA with post-hoc Tukey HSD Test) for each group. Groups with different letters indicate significant differences between groups, and groups with the same letter means non-significant differences between groups.

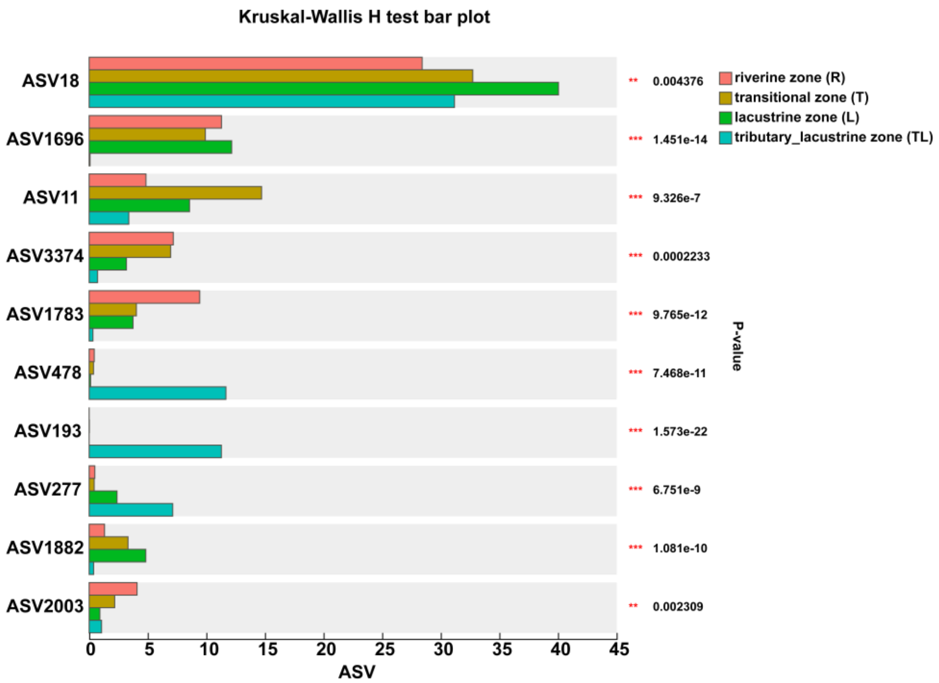

Figure S13. Kruskal-Wallis H test bar plot was used to detect MOB species with abundances ranked in top 10 (including sensitive MOB species) to assess the significance of the observed differences in the riverine zone (R), transitional zone (T), lacustrine zone (L) and tributary-lacustrine zone (TL).

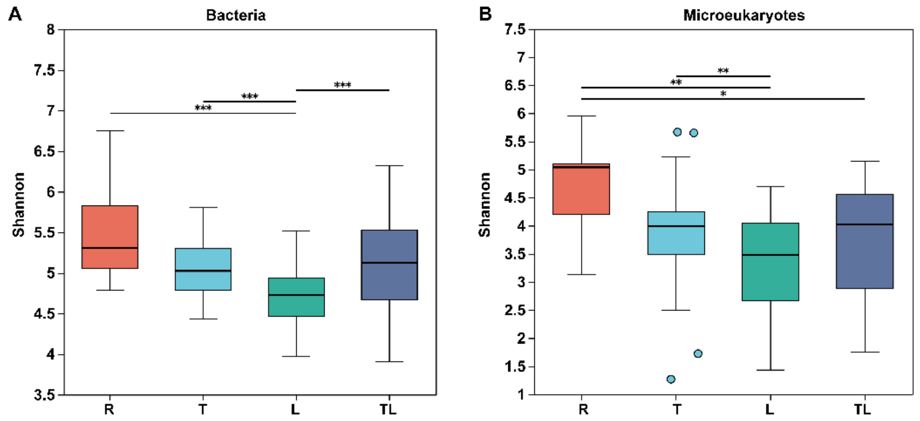

Figure S14. Comparison of Shannon index for bacteria and microeukaryotes in the riverine zone (R), transitional zone (T), lacustrine zone (L) and tributary-lacustrine zone (TL).

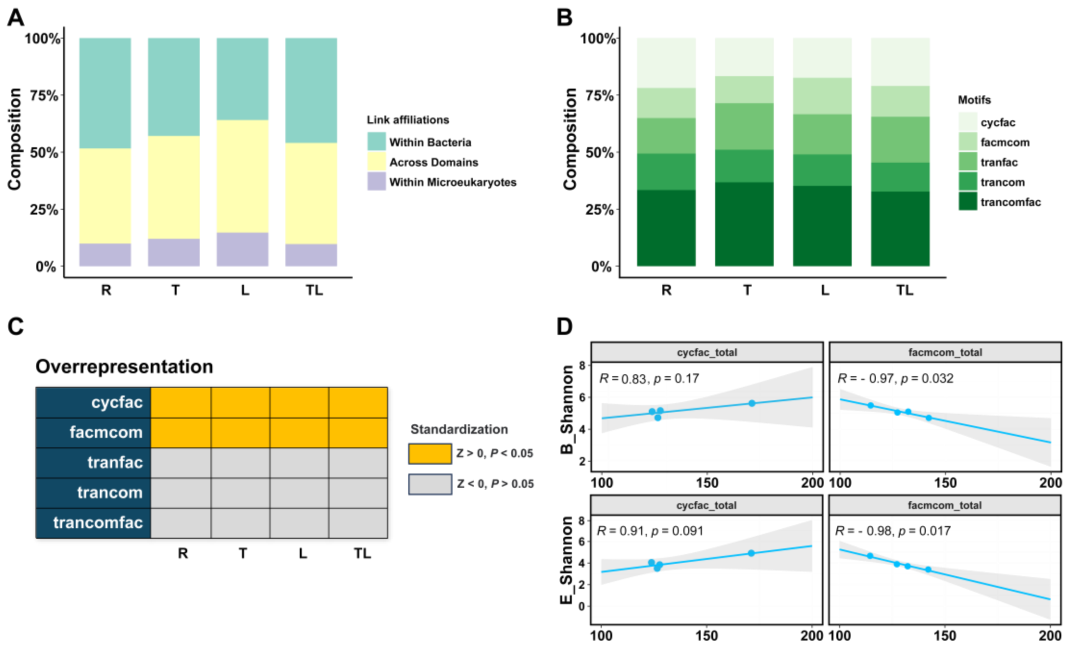

Figure S15. Network motifs within aquatic ecological networks of bacteria and microeukaryotes in the riverine zone (R), transitional zone (T), lacustrine zone (L), and tributary-lacustrine zone (TL). **A** Distribution of link affiliations for each ecological network, including within bacteria, within microeukaryotes, and across domains (i.e., across bacteria and microeukaryotes). **B** Composition bar plot of network motifs among triads of taxa detected in these four networks. Different colors indicated the types of identified motifs; cycfac, cycle facilitation; facmcom, facilitation-mediated competition; tranfac, transitive facilitation; trancom, transitive competition; trancomfac, transitive competition and facilitation. **C** Overrepresentation heatmap of network motifs. The importance of network motifs was assessed by the z-score of motifs relative to chance, thus acting as concern modules. Orange (Z > 0, *P* < 0.05) and brown (Z < 0, *P* > 0.05) blocks represent motifs that significantly occurred more and less than expected by chance, respectively. **D** Relationships between Shannon diversity of bacteria (B_Shannon) and microeukaryotes (E_Shannon) and overrepresentation of cycfac and facmcom motifs.

Supplementary Tables

Table S1 Sampling site location information

Sampling site		Coordinate		Distance to the dam (km)	Width surface (km)	Depth (m)
XLD	Shibantan (L2)	N 28°08' 43.69"	E 103°30' 12.62"	20	0.55	192
	Yanziyan (L1)	N 28°14' 46.83"	E 103°37' 05.62"	2	0.69	200
XJB	Yongshanqiao (B7)	N 28°14' 19.25"	E 103°40' 40.68"	140	0.14	30
	Huixizhen (B6)	N 28°18' 41.58"	E 103°52' 37.71"	110	0.28	45
	Xiaheba (B5)	N 28°28' 00.09"	E 103°49' 36.34"	95	0.25	68
	Suijiangxian (B4)	N 28°35' 59.09"	E 103°56' 46.75"	50	1.44	90
	Huilongcun (B3)	N 28°37' 43.35"	E 104°08' 48.18"	30	1.34	123
	Sanwangou (B2)	N 28°37' 56.66"	E 104°16' 16.63"	15	0.75	110
	Xintanba (B1)	N 28°38' 53.03"	E 104°22' 46.80"	2	0.92	117
	Shuifudaqiao (X1)	N 28°37' 59.79"	E 104°24' 54.51"	1.5	0.15	30
TGR	Zhutuo (ZT)	N 29°00' 50.18"	E 105°51'24.68"	730	0.69	6
	Mudong (MD)	N 29°34' 41.44"	E 106°50'31.81"	550	0.73	44
	Fuling (FL)	N 29°51' 41.54"	E 107°31'50.92"	450	0.96	56
	Zhongxian (ZX)	N 30°24'53.87"	E 108°10'58.55"	330	1.08	69
	Wanzhou (WZ)	N 30°54'20.07"	E 108°31'39.61"	260	0.99	48
	Gaoyang1 (GY1)	N 31°05'41.08"	E 108°40'27.59"	275	1.00	48
	Gaoyang2 (GY2)	N 31°06'09.06"	E 108°40'44.20"	277	0.24	83
	Yunyang (YY)	N 30°57'01.96"	E 108°54'44.04"	220	0.63	109
	Wushan (WS)	N 31°03'19.43"	E 109°51'26.80"	120	0.92	121
	Zigui (ZG)	N 30°50'05.73"	E 111°04'02.79"	2	2.95	130
	Sandouping (SDP)	N 30°50'05.73"	E 111°04'02.79"	10	0.71	50
SZT	Shizitan3 (SZT3)	N 29°57'53.77"	E 107°17'2.12"	12	0.31	19
	Shizitan2 (SZT2)	N 29°55'54.50"	E 107°17'27.37"	5	1.15	21
	Shizitan1 (SZT1)	N 29°54'59.06"	E 107°15'27.06"	1.5	2.31	30

Table S2 The concentration of POC, DOC (mg/L) and their isotopes (δ^{13}C, ‰)

Sample	POC	DOC	POC(δ^{13}C)	DOC(δ^{13}C)	α(POC-DOC)	ε(POC-DOC)
BYT1_J_d	0.230309	2.462	-23.63	-28.42	1.00493	4.930114
BYT1_M_d	2.205481	2.8555	-25.58	-23.63	0.998003	-1.99719
BYT1_N_d	0.425871	1.8445	-29.31	-28.21	0.998868	-1.13193
BYT2_J_d	0.59806	2.568	-26.88	-28.32	1.001482	1.481969
BYT2_M_d	1.900498	2.981	-25.91	-25.75	0.999836	-0.16423
BYT2_N_d	0.324939	1.698	-28.8	-28.7	0.999897	-0.10295
July_1	0.234537	1.2735	-29.27	-26.64	0.997298	-2.70198
July_10	0.180887	1.043	-27.99	-26.93	0.998911	-1.08934
July_11	0.248034	2.133	-25.72	-27.15	1.00147	1.469908
July_12	0.592577	2.3645	-26.61	-27.48	1.000895	0.894583
July_13	0.373946	2.0195	-26.4	-26.24	0.999836	-0.16431
July_14	0.332992	1.996	-25.91	-26.98	1.0011	1.099669
July_15	0.308891	1.896	-26.16	-27.32	1.001193	1.192581
July_16	0.298441	1.872	-26.93	-27.48	1.000566	0.565541
July_17	0.29151	1.795	-27.01	-28.02	1.001039	1.039116
July_18	0.15837	1.6055	-27.6	-28.01	1.000422	0.421815
July_19	0.390289	1.747	-26.31	-27.39	1.00111	1.110414
July_2	0.18489	1.261	-27.83	-27.06	0.999209	-0.79142
July_20	1.592791	3.724	-25.58	-27.88	1.002366	2.365963
July_21	1.135549	3.665	-24.66	-27.58	1.003003	3.002818
July_24	1.934641	4.386	-30.28	-28.92	0.998599	-1.4005
July_25	1.868004	4.3785	-30.36	-28.52	0.998106	-1.89402
July_26	1.453455	4.4485	-32.87	-29.21	0.99623	-3.77013
July_3	0.153379	1.4005	-28.48	-26.22	0.997679	-2.32085
July_4	0.203432	1.1275	-27.58	-26.85	0.99925	-0.75014
July_5	0.190296	1.1535	-27.43	-26.12	0.998655	-1.34513
July_6	0.164324	1.1755	-27.86	-26.82	0.998931	-1.06866

July_7	0.240284	1.3885	-28.4	-25.83	0.997362	-2.63814
July_8	0.214918	1.152	-28.18	-26.24	0.998008	-1.99228
July_9	0.339831	1.1005	-29.13	-26.57	0.99737	-2.62988
LCS1_J_d	0.739499	3.763	-31.46	-29.25	0.997723	-2.27659
LCS1_M_d	0.973594	4.3775	-28.45	-29.6	1.001185	1.185078
LCS1_N_d	0.284909	3.761	-33.56	-28.07	0.994351	-5.64855
LCS2_J_d	0.844453	4.028	-31.63	-29.15	0.997446	-2.55446
LCS2_M_d	1.397028	4.603	-27.13	-30.47	1.003445	3.444968
LCS2_N_d	0.282356	3.61	-32.57	-28.14	0.995442	-4.55827
LCS3_J_d	1.189376	4.166	-32.15	-28.46	0.996202	-3.79809
LCS3_M_d	0.867601	4.2555	-26.74	-30.33	1.003702	3.70229
LCS3_N_d	0.306474	3.5805	-32.23	-27.97	0.995617	-4.38258
May_1	0.212302	1.5675	-26.99	-26.17	0.999158	-0.84204
May_10	0.21228	1.373	-26.11	-27.2	1.00112	1.120477
May_11	0.210837	1.1445	-26.64	-33.22	1.006806	6.806099
May_12	0.349737	1.3895	-25.34	-32.09	1.006974	6.973789
May_13	0.269001	1.0625	-27.04	-30.85	1.003931	3.93128
May_14	0.222662	1.179	-27.71	-31.96	1.00439	4.390314
May_15	0.255352	1.8625	-29.64	-33.45	1.003942	3.941855
May_16	0.216533	2.0025	-28.97	-31.07	1.002167	2.167339
May_17	0.159811	1.583	-29.22	-31.71	1.002572	2.571544
May_18	0.167745	1.4975	-28.6	-31.87	1.003378	3.377646
May_19	0.076003	1.6395	-27.52	-31.83	1.004452	4.451698
May_2	0.195552	1.774	-28.51	-27.17	0.998623	-1.37742
May_20	1.653282	2.9145	-26.97	-27.14	1.000175	0.174743
May_21	2.378988	2.9015	-22.62	-27.64	1.005163	5.162697
May_24	1.509979	4.8775	-30.44	-30.28	0.999835	-0.165
May_25	1.759807	4.6685	-27.19	-29.23	1.002101	2.101425
May_26	1.831517	4.64	-25.75	-29.43	1.003792	3.791586

May_3	0.200313	1.2315	-27.22	-26.85	0.99962	-0.38021
May_4	0.16225	1.636	-27.03	-27.02	0.99999	-0.01028
May_5	0.221214	1.3895	-26.32	-26.58	1.000267	0.2671
May_6	0.224405	1.3315	-28.73	-27.19	0.998417	-1.58304
May_7	0.187598	1.4355	-27.71	-27.62	0.999907	-0.09256
May_8	0.337804	1.4485	-30.93	-26.96	0.99592	-4.08
May_9	0.189616	1.4775	-26.92	-27.8	1.000905	0.905164
Nov_1	0.063091	0.8665	-25.52	-25.72	1.000205	0.20528
Nov_10	0.06056	0.6095	-24.77	-27.39	1.002694	2.693783
Nov_11	0.307852	0.8275	-25.72	-27.12	1.001439	1.439026
Nov_12	0.259651	1.5535	-25.96	-27.2	1.001275	1.274671
Nov_13	0.129276	1.2595	-26.97	-27.19	1.000226	0.226149
Nov_14	0.16651	1.102	-26.9	-27.18	1.000288	0.287823
Nov_15	0.194075	1.0435	-27.13	-27.53	1.000411	0.411324
Nov_16	0.129445	1.2035	-27.46	-27.8	1.00035	0.349722
Nov_17	0.134386	1.2485	-27.11	-26.79	0.999671	-0.32881
Nov_18	0.140986	1.179	-27.65	-27.82	1.000175	0.174865
Nov_19	0.159265	0.9395	-27.59	-29.68	1.002154	2.153929
Nov_2	0.082444	0.448	-23.06	-27.69	1.004762	4.761856
Nov_20	0.345222	1.8215	-31.78	-28.85	0.996983	-3.01704
Nov_21	0.339407	1.797	-30.48	-28.56	0.998024	-1.97645
Nov_24	0.428412	3.815	-33.01	-28.06	0.994907	-5.09291
Nov_25	0.338758	3.6485	-32.96	-28.01	0.994907	-5.09264
Nov_26	0.296774	3.8325	-32.01	-27.91	0.995782	-4.21772
Nov_3	0.102214	0.642	-25.57	-28.53	1.003047	3.046929
Nov_4	0.092826	0.7065	-25.97	-28.8	1.002914	2.913921
Nov_5	0.100029	0.6115	-25.15	-28.44	1.003386	3.386307
Nov_6	0.121803	0.848	-25.8	-27.5	1.001748	1.748072
Nov_7	0.111747	0.31	-26.21	-27.02	1.000832	0.832494

Nov_8	0.084024	0.5995	-25.74	-27.21	1.001511	1.511118
Nov_9	0.063946	0.5085	-24.71	-27.09	1.002446	2.446269
RJS5_J_d	0.27164	1.13	-27.74	-26.42	0.998644	-1.35582
RJS5_M_d	0.187278	1.3835	-26.63	-26.64	1.00001	0.010274
RJS5_N_d	0.089346	0.48	-25.51	-28.57	1.00315	3.149995
RJS6_J_d	0.228551	1.239	-27.94	-26.13	0.998141	-1.85856
RJS6_J_z	0.203286	1.5055	-27.77	-25.98	0.998162	-1.83774
RJS6_M_d	0.191702	1.482	-26.19	-28.33	1.002202	2.202394
RJS6_M_z	0.314601	1.173	-27.02	-26.54	0.999507	-0.49309
RJS6_N_d	0.119022	0.4615	-25.74	-27.31	1.001614	1.614081
RJS6_N_z	0.083104	0.516	-25.76	-27.3	1.001583	1.583222
RJS7_J_d	0.261157	1.604	-27.18	-25.69	0.998471	-1.52929
RJS7_J_z	0.223594	1.4265	-27.99	-26.01	0.997967	-2.03288
RJS7_M_d	0.254966	1.882	-25.76	-26.89	1.001161	1.161225
RJS7_M_z	0.225709	1.482	-27.13	-28.2	1.001101	1.10105
RJS7_N_d	0.083117	0.4445	-26.17	-27.34	1.001203	1.202887
RJS7_N_z	0.101395	0.4235	-26.41	-26.95	1.000555	0.554956
RYT2_M_d	0.282876	1.5835	-26.24	-32.12	1.006075	6.075133
RYT2_N_d	0.204014	1.4625	-25.98	-26.37	1.000401	0.400563
RYT3_M_d	0.3842	1.282	-26.43	-31.55	1.005287	5.286798
RYT3_N_d	0.160267	1.2815	-26.94	-26.25	0.999291	-0.7086
RYT4_J_d	0.474688	1.835	-25.87	-27.54	1.001717	1.717294
RYT4_J_z	0.310658	1.8855	-25.79	-27.57	1.00183	1.830466
RYT4_M_d	0.209847	1.113	-27.77	-31.71	1.004069	4.069029
RYT4_M_z	0.231883	1.529	-27.34	-32.21	1.005032	5.032083
RYT4_N_d	0.153337	1.3435	-27.67	-27.36	0.999681	-0.31872
RYT4_N_z	0.151136	1.238	-27.03	-27.39	1.00037	0.370138
RYT5_J_d	0.368184	1.8855	-26.16	-26.24	1.000082	0.082156
RYT5_J_z	0.262844	2.014	-26.23	-27.32	1.001121	1.120615

RYT5_M_d	0.316285	2.109	-28.5	-32.1	1.003719	3.719392
RYT5_M_z	0.255373	1.8895	-28.83	-30.78	1.002012	2.011927
RYT5_N_d	0.161006	1.2375	-27.09	-28.63	1.001585	1.58539
RYT5_N_z	0.159314	1.1715	-27.12	-29.07	1.002008	2.008384
RYT6_J_d	0.42294	2.0905	-26.65	-28.01	1.001399	1.399191
RYT6_J_z	0.365268	1.878	-26.14	-27.72	1.001625	1.625046
RYT6_M_d	0.221237	1.941	-29.32	-30.83	1.001558	1.558034
RYT6_M_z	0.209747	1.639	-29.55	-31.19	1.001693	1.692798
RYT6_N_d	0.138405	1.2585	-27.22	-27.32	1.000103	0.102809
RYT6_N_z	0.160018	1.163	-27.34	-25.97	0.998593	-1.40653
TGD_1b2_11	0.165954	1.482	-27.39	-27.75	1.00037	0.370275
TGD_1b2_5	0.18424	1.799	-27.45	-31.75	1.004441	4.441002
TGD_1b2_7	0.316475	1.3955	-26.22	-28.06	1.001893	1.893121
TGD_1b3_11	0.140715	1.1485	-27.61	-28.26	1.000669	0.668903
TGD_1b3_5	0.216894	1.791	-27.5	-30.82	1.003426	3.425576
TGD_1b3_7	0.339552	1.702	-26.7	-27.67	1.000998	0.997604
TGD_2b2_11	0.156115	1.1035	-27.91	-29.71	1.001855	1.855115
TGD_2b2_5	0.145578	1.455	-27.57	-30.08	1.002588	2.587842
TGD_2b2_7	0.320757	1.672	-27.4	-28.01	1.000628	0.627578
TGD_2b3_11	0.158585	1.1645	-27.75	-27.76	1.00001	0.010286
TGD_2b3_5	0.147292	1.3125	-27.82	-31.72	1.004028	4.027761
TGD_2b3_7	0.36197	1.6815	-27.32	-27.51	1.000195	0.195375
TGD_2b4_11	0.132457	1.25	-27.39	-27.75	1.00037	0.370275
TGD_2b4_5	0.132576	1.4685	-27.78	-31.65	1.003996	3.996489
TGD_2b4_7	0.381099	1.653	-26.9	-27.66	1.000782	0.78162
TGD_2b5_11	0.166563	1.0225	-27.61	-27.75	1.000144	0.143996
TGD_2b5_5	0.170174	1.5635	-27.25	-31.33	1.004212	4.211961
TGD_2b5_7	0.404342	1.417	-26.93	-27.88	1.000977	0.977246
XJB_1b2_11	0.084808	0.877	-25.77	-27.78	1.002067	2.067433

XJB_1b2_5	0.469016	1.5995	-30.37	-26.74	0.99627	-3.72973
XJB_1b2_7	0.20712	1.117	-27.45	-26.71	0.99924	-0.76031
XJB_1b3_11	0.154649	1.0505	-24.86	-25.74	1.000903	0.90325
XJB_1b3_5	0.219657	1.631	-29.12	-26.44	0.997247	-2.75278
XJB_1b3_7	0.177065	1.254	-27.35	-27.06	0.999702	-0.29807
XJB_2b2_11	0.063064	0.613	-26.2	-27.57	1.001409	1.408842
XJB_2b2_5	0.265331	1.318	-26.43	-26.44	1.00001	0.010272
XJB_2b2_7	0.181883	1.319	-27.1	-27.09	0.99999	-0.01028
XJB_2b3_11	0.080923	0.5565	-25.74	-27.16	1.00146	1.459644
XJB_2b3_5	0.168546	1.4855	-28.75	-26.23	0.997412	-2.58788
XJB_2b3_7	0.244232	1.3505	-28.01	-26.63	0.998582	-1.41775
XJB_2b4_11	0.064668	0.4765	-26.47	-27.31	1.000864	0.863584
XJB_2b4_5	0.255738	1.4455	-28.29	-26.03	0.99768	-2.3204
XJB_2b4_7	0.193323	1.344	-26.78	-26.87	1.000092	0.092485
XJB_2b5_11	0.068544	0.966	-26	-27.05	1.001079	1.079192
XJB_2b5_5	0.172209	1.274	-27.26	-27	0.999733	-0.26721
XJB_2b5_7	0.174143	1.2025	-27.27	-26.99	0.999712	-0.28777
XLD_1b2_11	0.078001	0.6615	-25.74	-28.74	1.003089	3.088771
XLD_1b2_5	0.178959	1.7375	-26.26	-25.74	0.999466	-0.53374
XLD_1b2_7	0.142161	1.0445	-27	-27.09	1.000093	0.092506
XLD_1b3_11	0.098528	1.0455	-25.74	-28.31	1.002645	2.644876
XLD_1b3_5	0.230019	1.469	-26.05	-27.64	1.001635	1.635197
XLD_1b3_7	0.259924	1.3575	-27.59	-26.92	0.999311	-0.68854
XLD_2b2_11	0.091809	0.509	-25.77	-28.14	1.002439	2.438623
XLD_2b2_5	0.210506	1.7405	-28.21	-28.35	1.000144	0.144085
XLD_2b2_7	0.196232	1.1385	-26.91	-26.56	0.99964	-0.35955
XLD_2b3_11	0.132144	0.7605	-25.45	-28.54	1.003181	3.180779
XLD_2b3_5	0.232025	1.4215	-29	-27.16	0.998109	-1.89137
XLD_2b3_7	0.214236	1.254	-28.3	-27.11	0.998777	-1.22316

XLD_2b4_11	0.110905	0.8205	-25.48	-28.47	1.003078	3.07762
XLD_2b4_5	0.186209	1.3515	-26.91	-28.33	1.001461	1.461402
XLD_2b4_7	0.231983	1.1645	-27.27	-27.1	0.999825	-0.17474
XLD_2b5_11	0.085575	0.4955	-25.55	-28.64	1.003181	3.181107
XLD_2b5_5	0.18656	1.436	-26.49	-28.64	1.002213	2.213392
XLD_2b5_7	0.201376	0.9715	-27.04	-26.98	0.999938	-0.06166

References

1. Raymond, P. A.; Spencer, R. G., riverine DOM. In *Biogeochemistry of marine dissolved organic matter*, Elsevier: 2015; pp 509-533.
2. Pohlman, J. W.; Bauer, J. E.; Waite, W. F.; Osburn, C. L.; Chapman, N. R., Methane hydrate-bearing seeps as a source of aged dissolved organic carbon to the oceans. *Nat. Geosci.* **2011,** *4*, (1), 37-41.
3. Zhang, C. L.; Dang, H. Y.; Azam, F.; Benner, R.; Legendre, L.; Passow, U.; Polimene, L.; Robinson, C.; Suttle, C. A.; Jiao, N. Z., Evolving paradigms in biological carbon cycling in the ocean. *Natl. Sci. Rev.* **2018,** *5*, (4), 481-499.
4. Fry, B., *Stable isotope ecology*. Springer: 2006; Vol. 521.
5. Brankovits, D.; Pohlman, J. W.; Niemann, H.; Leigh, M. B.; Leewis, M. C.; Becker, K. W.; Iliffe, T. M.; Alvarez, F.; Lehmann, M. F.; Phillips, B., Methane- and dissolved organic carbon-fueled microbial loop supports a tropical subterranean estuary ecosystem. *Nat. Commun.* **2017,** *8*, 12.
6. Summons, R. E.; Jahnke, L. L.; Roksandic, Z., Carbon isotopic fractionation in lipids from methanotrophic bacteria - relevance for interpretation of the geochemical record of biomarkers. *Geochim. Cosmochim. Acta* **1994,** *58*, (13), 2853-2863.
7. Faust, K.; Raes, J., Microbial interactions: from networks to models. *Nat. Rev. Microbiol.* **2012,** *10*, (8), 538-550.
8. Ma, B.; Wang, Y.; Ye, S.; Liu, S.; Stirling, E.; Gilbert, J. A.; Faust, K.; Knight, R.; Jansson, J. K.; Cardona, C.; Rottjers, L.; Xu, J., Earth microbial co-occurrence network reveals interconnection pattern across microbiomes. *Microbiome* **2020,** *8*, (1), 82.
9. Xu, Z.; Woodhouse, J. N.; Te, S. H.; Yew-Hoong Gin, K.; He, Y.; Xu, C.; Chen, L., Seasonal variation in the bacterial community composition of a large estuarine reservoir and response to cyanobacterial proliferation. *Chemosphere* **2018,** *202*, 576-585.
10. Kurtz, Z. D.; Muller, C. L.; Miraldi, E. R.; Littman, D. R.; Blaser, M. J.; Bonneau, R. A., Sparse and Compositionally Robust Inference of Microbial Ecological Networks. *PLoS Comput. Biol.* **2015,** *11*, (5), 25.
11. Hu, A.; Ju, F.; Hou, L.; Li, J.; Yang, X.; Wang, H.; Mulla, S. I.; Sun, Q.; Burgmann, H.; Yu, C. P., Strong impact of anthropogenic contamination on the co-occurrence patterns of a riverine microbial community. *Environ. Microbiol.* **2017,** *19*, (12), 4993-5009.
12. Milo, R.; Shen-Orr, S.; Itzkovitz, S.; Kashtan, N.; Chklovskii, D.; Alon, U., Network motifs: Simple building blocks of complex networks. *Science* **2002,** *298*, (5594), 824-827.
13. Liu, X.; Chu, H.; Godoy, O.; Fan, K.; Gao, G.-F.; Yang, T.; Ma, Y.; Delgado-Baquerizo, M., Positive associations fuel soil biodiversity and ecological networks worldwide. *Proc. Natl. Acad. Sci. U. S. A.*

2024, *121*, (6), e2308769121.

14. Losapio, G.; Schöb, C.; Staniczenko, P. P. A.; Carrara, F.; Palamara, G. M.; De Moraes, C. M.; Mescher, M. C.; Brooker, R. W.; Butterfield, B. J.; Callaway, R. M.; Cavieres, L. A.; Kikvidze, Z.; Lortie, C. J.; Michalet, R.; Pugnaire, F. I.; Bascompte, J., Network motifs involving both competition and facilitation predict biodiversity in alpine plant communities. *Proc. Natl. Acad. Sci. U. S. A.* **2021,** *118*, (6), 6.

15. Song, C. L.; Simmons, B. I.; Fortin, M. J.; Gonzalez, A.; Kaiser-Bunbury, C. N.; Saavedra, S., Rapid monitoring of ecological persistence. *Proc. Natl. Acad. Sci. U. S. A.* **2023,** *120*, (20), 10.

16. Xun, W. B.; Liu, Y. P.; Li, W.; Ren, Y.; Xiong, W.; Xu, Z. H.; Zhang, N.; Miao, Y. Z.; Shen, Q. R.; Zhang, R. F., Specialized metabolic functions of keystone taxa sustain soil microbiome stability. *Microbiome* **2021,** *9*, (1), 15.

17. Shi, Y.; Zhang, K. P.; Li, Q.; Liu, X.; He, J. S.; Chu, H. Y., Interannual climate variability and altered precipitation influence the soil microbial community structure in a Tibetan Plateau grassland. *Sci. Total Environ.* **2020,** *714*, 8.

18. Wu, J.; Barahona, M.; Tan, Y. J.; Deng, H. Z., Natural Connectivity of Complex Networks. *Chin. Phys. Lett.* **2010,** *27*, (7), 4.

19. Herren, C. M.; McMahon, K. D., Cohesion: a method for quantifying the connectivity of microbial communities. *ISME J.* **2017,** *11*, (11), 2426-2438.

20. Stegen, J. C.; Lin, X. J.; Fredrickson, J. K.; Chen, X. Y.; Kennedy, D. W.; Murray, C. J.; Rockhold, M. L.; Konopka, A., Quantifying community assembly processes and identifying features that impose them. *ISME J.* **2013,** *7*, (11), 2069-2079.

21. Lewins, R., Evolution in Changing Environments: Some Theoretical Explorations. In Princeton University Press: 1968.

22. Jiao, S.; Yang, Y.; Xu, Y.; Zhang, J.; Lu, Y., Balance between community assembly processes mediates species coexistence in agricultural soil microbiomes across eastern China. *ISME J.* **2020,** *14*, (1), 202-216.

23. Liaw, A.; Wiener, M., Classification and regression by randomForest. *R News* **2002,** *2*, (3), 18-22.

24. Zeileis, A.; Leisch, F.; Hornik, K.; Kleiber, C., strucchange: An R package for testing for structural change in linear regression models. *J. Stat. Softw.* **2002,** *7*, 1-38.

25. de Vries, F. T.; Griffiths, R. I.; Bailey, M.; Craig, H.; Girlanda, M.; Gweon, H. S.; Hallin, S.; Kaisermann, A.; Keith, A. M.; Kretzschmar, M.; Lemanceau, P.; Lumini, E.; Mason, K. E.; Oliver, A.; Ostle, N.; Prosser, J. I.; Thion, C.; Thomson, B.; Bardgett, R. D., Soil bacterial networks are less stable under drought than fungal networks. *Nat. Commun.* **2018,** *9*, (1), 3033.

26. Tang, Q.; Lu, L. H.; Luo, F.; Li, X. R.; Zhang, Y. Y.; Li, R.; Bernal, C.; Vera, S.; Izaguirre, I.; Xiao, Y.; Li, Z., Terrigenous organic carbon contributes to reservoir carbon emissions: Potential role of the microbial community along a trophic gradient. *J. Hydrol.* **2023,** *621*, 11.
